# Chiral and Polar Duality Design of Heteroanionic Compounds: Sr_18_Ge_9_O_5_S_31_ Based on [Sr_3_OGeS_3_]^2+^ and [Sr_3_SGeS_3_]^2+^ Groups

**DOI:** 10.1002/advs.202306825

**Published:** 2023-12-08

**Authors:** Shaoxin Cui, Hongping Wu, Xinkang Dong, Zhanggui Hu, Jiyang Wang, Yicheng Wu, Kenneth R. Poeppelmeier, Hongwei Yu

**Affiliations:** ^1^ Tianjin Key Laboratory of Functional Crystal Materials Institute of Functional Crystal, College of Materials Science and Engineering Tianjin University of Technology Tianjin 300384 China; ^2^ Department of Chemistry Northwestern University Evanston IL 60208 USA

**Keywords:** chiral and polar duality, heteroanionic materials, hourglass‐like groups, NLO, symmetry breaking

## Abstract

Chirality and polarity are the two most important and representative symmetry‐dependent properties. For polar structures, all the twofold axes perpendicular to the principal axis of symmetry should be removed. For chiral structures, all the mirror‐related symmetries and inversion axes should be removed. Especially for duality (polarity and chirality), all of the above symmetries should be broken and that also represents the highest‐level challenge. Herein, a new symmetry‐breaking strategy that employs heteroanionic groups to construct hourglass‐like [Sr_3_OGeS_3_]^2+^ and [Sr_3_SGeS_3_]^2+^ groups to design and synthesize a new oxychalcogenide Sr_18_Ge_9_O_5_S_31_ with chiral‐polar duality is proposed. The presence of two enantiomers of Sr_18_Ge_9_O_5_S_31_ is confirmed by the single‐crystal X‐ray diffraction. Its optical activity and ferroelectricity are also studied by solid‐state circular dichroism spectroscopy and piezoresponse force microscopy, respectively. Further property measurements show that Sr_18_Ge_9_O_5_S_31_ possesses excellent nonlinear optical properties, including the strong second harmonic generation efficiency (≈2.5 × AGS), large bandgap (3.61 eV), and wide mid‐infrared transparent region (≈15.3 µm). These indicate that the unique microstructure groups of heteroanionic materials are conducive to realizing symmetry‐breaking and are able to provide some inspiration for exploring the chiral‐polar duality materials.

## Graphical abstract

A new oxychalcogenide Sr_18_Ge_9_O_5_S_31_ with chiral‐polar duality is successfully designed and synthesized by employing heteroanionic groups to construct hourglass‐like [Sr_3_OGeS_3_]^2+^ and [Sr_3_SGeS_3_]^2+^ groups. And owing to the chiral‐polar duality, Sr_18_Ge_9_O_5_S_31_ can exhibit optical activity, ferroelectric, and nonlinear optical properties.

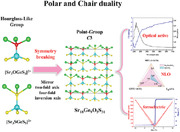

## 1 Introduction

**Figure 1:**
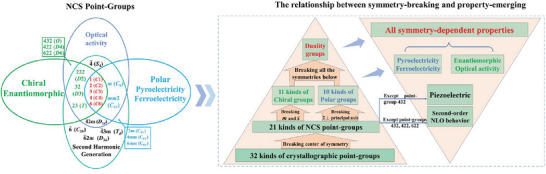
The NCS point‐groups^[^ ^2a^ ^]^ and the relationship between symmetry‐breaking and property‐emerging.

Symmetry of crystal structure serves as the fundamental basis for comprehending the material's properties, encompassing not only its macroscopic shape but also its physical attributes, such as electrical and optical characteristics. Especially, breaking symmetry can make structures obtain some important and interesting functional properties.^[^ ^1^ ^]^ For example, removing the center of symmetry can achieve a non‐centrosymmetric (NCS) structure. In all the NCS point groups, except 432 (*O*) point group, interesting piezoelectricity can be obtained, and further excluding 422 (*D*_4_) and 622 (*D*_6_) point groups, interesting second‐order nonlinear optical (NLO) effects can be obtained.^[^ ^2^ ^]^ Moreover, when the twofold axes perpendicular to the principal axis of symmetry are removed, polarity emerges, resulting in new pyroelectric and ferroelectric properties.^[^ ^3^ ^]^ Similarly, when removing the mirrors and inversion axes can make materials crystallize in chiral point groups, which can exhibit more enantiomorphic, circular dichroism (CD), and optically active properties.^[^ ^4^ ^]^ The relationship between symmetry‐breaking and property‐emerging can be described by **Figure** **1**. Clearly, when more types of symmetry elements are broken, more functional properties can be obtained. Especially when all the center of symmetry, the twofold axis perpendicular to the principal axis of symmetry, the mirror symmetry, and inversion axis symmetry are broken, materials will be able to exhibit chiral‐polar duality and obtain all the symmetry‐dependent properties, including piezoelectricity, second‐order NLO behavior, ferroelectricity, pyroelectricity, enantiomorphic, and optical activity. Obviously, realizing this feat is the most difficult task, as only the compounds crystallizing in the point groups of 1 (*C*_1_), 2 (*C*_2_), 3 (*C*_3_), 4 (*C*_4_), and 6 (*C*_6_) are possible to break all the above symmetries and exhibit all the symmetry‐dependent functional properties. Although, many effective strategies for the synthesis of polar or chiral compounds have been developed.^[^ ^5^ ^]^ However, the design strategies for duality compounds are still scarce. Recently, Wang et al. have conducted a comprehensive investigation on the breaking of inversion symmetry in crystalline racemates, suggesting that different types of racemic compounds are produced when the enantiomers exhibit distinct symmetries and emphasizing the importance of nonparallel packing of ligands.^[^ ^6^ ^]^ This study provides valuable insights for achieving NCS racemates by breaking the inversion symmetry between enantiomers.

In this research, we are interested in the heteroanionic compounds because they exhibit a rich structural chemistry and excellent functional properties, which have been highlighted in the recent review papers from Kageyama's and Rondinelli's groups.^[^ ^7^ ^]^ Especially for the NLO materials that can expand the spectral regions of solid‐state lasers by the second harmonic generation (SHG), heteroanionic compounds can exhibit more balanced NLO properties, as observed in AeGeOSe_2_ (Ae = Sr, Ba),^[^ ^8^ ^]^ Ae_2_GeGa_2_OS_6_ (Ae = Ca, Sr),^[^ ^9^ ^]^ Ae_3_Q[GeOQ_3_] (Ae = Sr, Ba; Q = S, Se),^[^ ^10^ ^]^ and Sr_3_[SnOSe_3_][CO_3_],^[^ ^11^ ^]^ for example. More importantly, heteroanionic compounds may also be an ideal materials class for achieving symmetry breaking because the difference of electronegativity, atomic radius, and coordination environments of the different types of anions may break the intrinsic symmetry of basic building units (BBUs). For example, for the single‐anionic MQ_4_ (M = Ga, In, Ge, Sn; Q = S, Se) tetrahedral groups, they typically exhibit the *T_d_* point‐group symmetry that includes threefold axis, twofold axis, fourfold inversion axis, and mirror symmetry. However, when a Q atom in the MQ_4_ tetrahedron is substituted by O atom to form the MOQ_3_ tetrahedron, the M─O distance tends to be shorter than M─Q distances owing to differences in radii and electronegativity.^[^ ^8b^ ^]^ This can result in the loss of symmetry such as the twofold axis and fourfold inversion axis, making the heteroanionic MOQ_3_ tetrahedron polar. Additionally, the O atom typically bonds to four cations, including the M cation at the center of the MOQ_3_ tetrahedron, and together with the MOQ_3_ tetrahedron forms hourglass‐like groups, N_3_OMQ_3_ (N = alkali or alkaline earth metals). Owing to steric hindrance, the atoms on the upper surface of the hourglass‐like group and the atoms on the lower surface of the hourglass‐like group don't align directly and instead rotate at a certain angle, leading to the breaking of the mirror symmetry of the tetrahedron and the emergence of chirality. As a result, the material may exhibit both chiral and polar duality. Similarly, the analogous phenomenon occurs when two or three Q atoms in the MQ_4_ tetrahedron are replaced by O atoms.

With these in mind, our focus is directed toward the oxychalcogenide system, where a series of excellent mid‐infrared (IR) NLO crystals have been synthesized.^[^ ^8^, ^9^, ^10^, ^11^, ^12^ ^]^ Also, in order to better maintain the local chiral and polar symmetry of heteroanionic hourglass‐like N_3_OMQ_3_ groups, we want to obtain their isolated anionic framework, i.e., forming the zero dimensional anionic structure through the “number effect” of cations. It is well‐known that the alkaline earth metal cations with low Lewis acid strengths can be used as the dimensional decomposition agent to facilitate the formation of isolated anion groups in the structures.^[^ ^13^ ^]^ By doing these, a new compound, Sr_18_Ge_9_O_5_S_31_ with the hourglass‐like [Sr_3_OGeS_3_]^2+^/[Sr_3_SGeS_3_]^2+^ groups has been successfully designed and synthesized. It crystallizes in the NCS and polar space group of *R*3 (No.146) and exhibits ferroelectricity as confirmed by piezoresponse force microscopy (PFM). Additionally, the SHG measurement shows that the SHG response of Sr_18_Ge_9_O_5_S_31_ is 2.5 times that of commercialized AgGaS_2_ (AGS). Also, it has a large bandgap (3.61 eV), and appropriate birefringence (Δ*n*) (0.026 @ 2090 nm). Remarkably, *R*3 is also one of the 65 Sohncke space groups capable of exhibiting important optical activity, as demonstrated by the solid‐state CD spectroscopy. Therefore, Sr_18_Ge_9_O_5_S_31_ is a rare and excellent compound with chirality and polarity properties. The synthetic method, crystal structure, and optical properties of Sr_18_Ge_9_O_5_S_31_ are stated in detail here. Theoretical calculations have been carried out to understand the structure‐property relationship.

## 2 Results and Discussion

### 2.1 Synthesis and Crystal Structure

**Figure 2:**
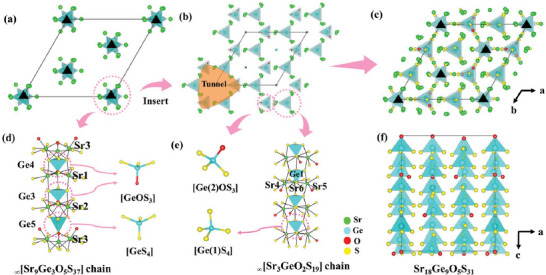
a) The _∞_[Sr_9_Ge_3_O_5_S_37_] chain located in the threefold axis positions; b) the _∞_[Sr_3_Ge_2_O_2_S_19_] framework composed of _∞_[Sr_3_GeO_2_S_19_] chain and Ge(2)OS_3_ tetrahedra located in the screw axis positions; c) the structure of Sr_18_Ge_9_O_5_S_31_ viewing along the c axis. d) _∞_[Sr_9_Ge_3_O_5_S_37_] chain; e) _∞_[Sr_3_GeO_2_S_19_] chain and Ge(2)OS_3_ tetrahedron; f) Ge‐based tetrahedra in aligned in unit cell viewing along the b axis. The S atoms in (a,b), Sr─S, and Sr─O bonds in (a–c) were omitted for clarity.

Sr_18_Ge_9_O_5_S_31_ was synthesized by the solid‐state reaction in sealed silica tubes. Its purity was confirmed by powder X‐ray diffraction (XRD) (Figure S1, Supporting Information). The structure of Sr_18_Ge_9_O_5_S_31_ was determined by the single crystal XRD, which demonstrates that Sr_18_Ge_9_O_5_S_31_ crystallizes in the NCS and polar rhombohedral space group, *R*3 (No. 146, Table S1, Supporting Information). The structure of Sr_18_Ge_9_O_5_S_31_ is shown in **Figure** **2**. Its asymmetric unit contains six Sr, five Ge, three O, and eleven S atoms. The Sr atoms are surrounded by the seven or eight O and S atoms with the Sr─O distances ranging from 2.422(8) to 2.631(12) Å and the Sr─S distances varying from 2.901(5) to 3.468(5) Å. Figure S2 in the Supporting Information also illustrates the complete coordination environments of Sr atoms. For Ge(1) and Ge (5) atoms, they are coordinated by four S atoms forming the single anionic [GeS_4_] tetrahedra with the Ge─S distances in the region of 2.186(7)−2.220(5) Å. For Ge(2), Ge(3), and Ge(4) atoms, they are connected by one O atom and three S atoms forming the distorted heteroanionic [GeOS_3_] tetrahedra with a short Ge─O distance of 1.770(2) Å and three long Ge─S distances in the region of 2.207(5)−2.227(5) Å. The selected bond distances and angles are listed in Table S2, Supporting Information. The bond valence calculations^[^ ^14^ ^]^ show that the bond valence sums for Sr, Ge, O, and S atoms are 1.76‐2.07, 3.92‐4.17, 1.94‐2.20, and 1.82‐2.14 (Table S3, Supporting Information), respectively, which are all consistent with their ideal oxidation states for each atom.

The structure of Sr_18_Ge_9_O_5_S_31_ is a complex 3D network composed of the _∞_[Sr_9_Ge_3_O_5_S_37_] chains positioned along the threefold axis and the _∞_[Sr_3_Ge_2_O_2_S_19_] framework situated on the screw axis (Figure 2a–c). The former determines the overall symmetry of the structure and consists of interconnected Sr(1)OS_6_, Sr(2)OS_7_, and Sr(3)OS_6_ polyhedra, which form [Sr_9_O_5_S_37_] clusters. Further, isolated Ge(3,4)OS_3_ and Ge(5)S_4_ tetrahedra are regularly arranged along *c* direction linked with the Sr(1‐3)‐based polyhedra to form _∞_[Sr_9_Ge_3_O_5_S_37_] chains (Figure 2d). The other part of the structure is composed of Ge(2)OS_3_ tetrahedra and _∞_[Sr_3_GeO_2_S_19_] chains that consist of the Sr(4)OS_7_, Sr(5)OS_6_, and Sr(6)S_8_ polyhedra and the Ge(1)S_4_ tetrahedra (Figure 2e) to form the _∞_[Sr_3_Ge_2_O_2_S_19_] framework with tunnels. Then the _∞_[Sr_9_Ge_3_O_5_S_37_] chains are located in the tunnels, which facilitates the uniform arrangement of the anionic groups in the voids (Figure 2f).

### 2.2 Symmetry Analysis

**Figure 3:**
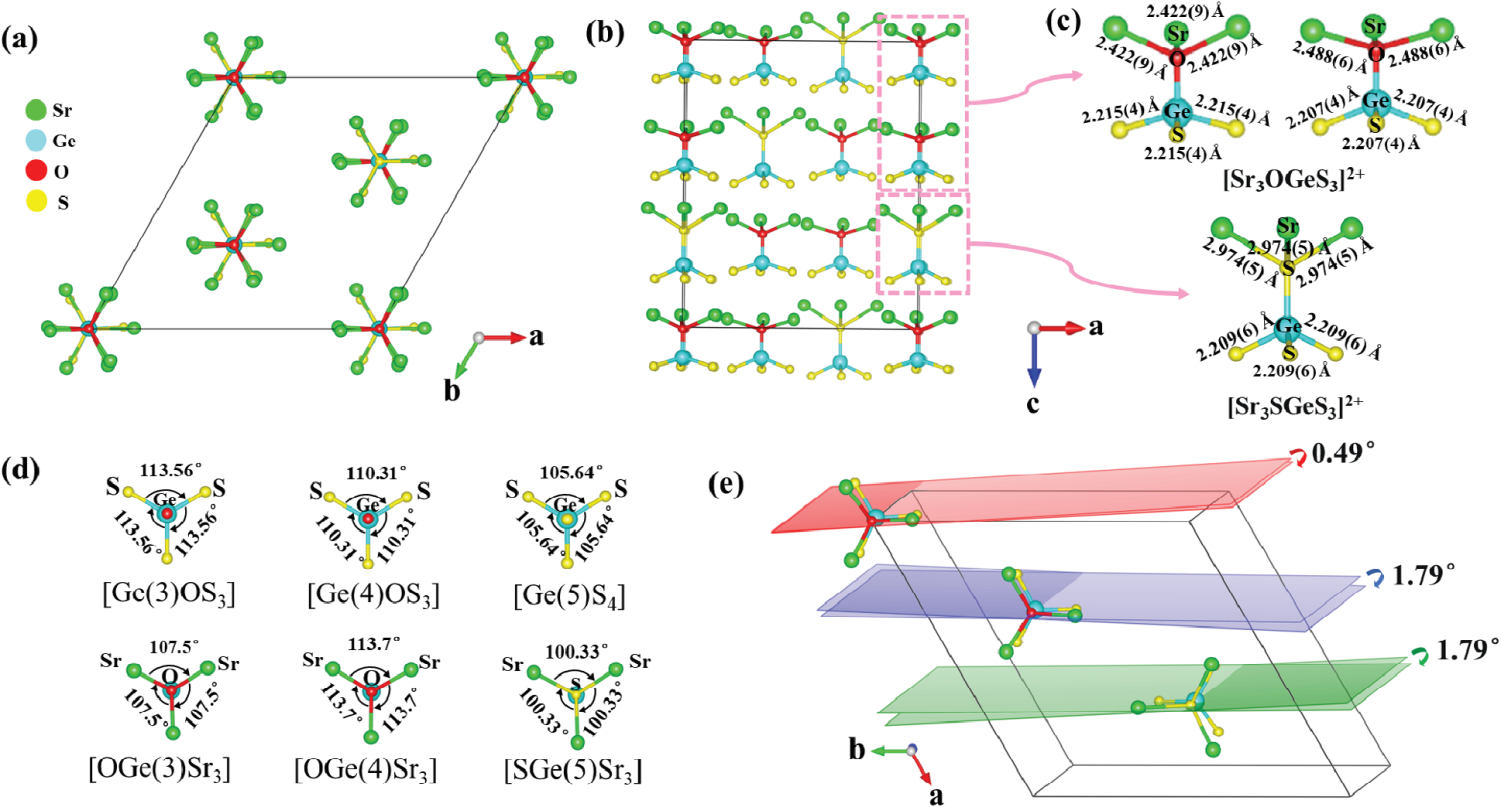
a) View the hourglass‐like groups on the threefold axes of Sr_18_Ge_9_O_5_S_31_ along *c* axis; b) view the hourglass‐like groups on the threefold axes of Sr_18_Ge_9_O_5_S_31_ along *b* axis; c) the isolated hourglass‐like groups [Sr_3_OGeS_3_]^2+^ and [Sr_3_SGeS_3_]^2+^; d) the bond angles in [Sr_3_OGeS_3_]^2+^ and [Sr_3_SGeS_3_]^2+^; e) the dihedral angles of [Sr_3_OGeS_3_]^2+^ and [Sr_3_SGeS_3_]^2+^ groups.

It is clear that the symmetry of Sr_18_Ge_9_O_5_S_31_ is mainly determined by the _∞_[Sr_9_Ge_3_O_5_S_37_] chains situated on the threefold axes, which imply that Sr_18_Ge_9_O_5_S_31_ crystallizes the trigonal crystal system. In fact, in the trigonal crystal systems, there are two lattice types, i.e., primitive (*P)* and rhombohedral *(R)* types of lattices. For Sr_18_Ge_9_O_5_S_31_, two distinct hourglass‐like groups, [Sr_3_OGeS_3_]^2+^ and [Sr_3_SGeS_3_]^2+^ have different volumes and they are staggered packed along c‐axis in the structure (**Figure** **3**a–c), which make the structure exhibit the *R‐*type Bravais lattices. Furthermore, it can be found that the threefold rotational symmetry of the Sr_18_Ge_9_O_5_S_31_ structure is also from their hourglass‐like [Sr_3_OGeS_3_]^2+^ and [Sr_3_SGeS_3_]^2+^ groups (Figure 3c,d). First, the [Sr_3_OGeS_3_]^2+^ and [Sr_3_SGeS_3_]^2+^ groups, are both constructed by the two different tetrahedra through the “top to top” connection. This connection breaks the symmetry of the fourfold inversion axis and twofold axis of their tetrahedra. In addition, as the non‐overlapping arrangements of two tetrahedra along c‐axis in the hourglass‐like groups, with the dihedral angles of 0.49° and 1.79° in planes consisting of Sr─O─Ge and O─Ge─S of [Sr_3_O(1)GeS_3_]^2+^ and [Sr_3_O(3)GeS_3_]^2+^, respectively, and the dihedral angle of 1.79° in the planes consisting of Sr─S─Ge and S─Ge─S of [Sr_3_SGeS_3_]^2+^ (Figure 3e), the mirror symmetry of their tetrahedra is also broken. That is, only the threefold rotational symmetry of the tetrahedra is retained in the hourglass‐like [Sr_3_OGeS_3_]^2+^ and [Sr_3_SGeS_3_]^2+^ groups. Furthermore, the aligned arrangements along *c*‐axis of these [Sr_3_OGeS_3_]^2+^ and [Sr_3_SGeS_3_]^2+^ groups make their local symmetry well appear in the structure, and result in the *R*3 symmetry. Therefore, the hourglass‐like [Sr_3_OGeS_3_]^2+^ and [Sr_3_SGeS_3_]^2+^ groups also have the main contribution to the macroscopic symmetry of the structure.

### 2.3 Chiral Properties

**Figure 4:**
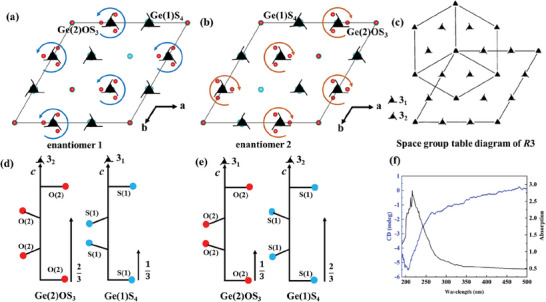
a,b) The single crystal structures of the two enantiomers of Sr_18_Ge_9_O_5_S_31_ exhibiting enantiomer 1 and enantiomer 2, respectively; c) the space group table diagram of *R*3; d) 3_2_ and 3_1_ screw axes are occupied by the counterclockwise rotating Ge(2)OS_3_ and clockwise rotating Ge(1)S_4_ groups in enantiomer 1, respectively; e) 3_1_ and 3_2_ screw axes are occupied by clockwise rotating Ge(2)OS_3_ and counterclockwise rotating Ge(1)S_4_ groups in enantiomer 2, respectively; f) the CD and corresponding UV−vis absorption spectra of Sr_18_Ge_9_O_5_S_31_ measured with the sample prepared through mixing pure polycrystalline with KBr at 1:150. All Sr and S atoms have been omitted for clarity.

Notably, the space group *R*3 is one of the 65 Sohncke space groups.^[^ ^2^, ^15^ ^]^ The crystals that are fabricated in these space groups always have a chiral structure. Therefore, several crystals were collected for structural determination in order to find two enantiomers of Sr_18_Ge_9_O_5_S_31_. The space group of *R*3 indicates that this space group may feature two different screw axis, 3_1_ or 3_2_ (**Figure** **4**a–c).^[^ ^16^ ^]^ After absolute structures were obtained from single‐crystal XRD with the flack parameter near 0, the handedness of the measured crystals could be determined by the type of anionic groups on the two screw axes. Fortunately, the pair of enantiomers of Sr_18_Ge_9_O_5_S_31_ are observed and crystal structures with enantiomer 1 and enantiomer 2 are shown in Figure 4a,b, respectively. Both enantiomers clearly show the 3_1_ and 3_2_ helicities featuring the Ge(2)OS_3_ and Ge(1)S_4_ tetrahedra. In the enantiomer 1, the Ge(2)OS_3_ tetrahedra exhibit 3_2_ symmetry, and Ge(1)S_4_ tetrahedra possess 3_1_ symmetry (Figure 4a,d). While in enantiomer 2, the phenomenon is exactly opposite, i.e., Ge(2)OS_3_ is located on 3_1_ symmetry, and Ge(1)S_4_ is located on 3_2_ symmetry (Figure 4b,e). In addition, we also observed the morphologies of the crystals in the two enantiomers as well as the polycrystalline sample by scanning electron microscopy (Figure S3, Supporting Information). But unfortunately, no significant difference was found. We also calculated their theoretical morphologies through the Mercury program based on the Bravais–Friedel–Donnay–Harker theory,^[^ ^17^ ^]^ which also suggests two enantiomers having similar morphologies (Figure S4, Supporting Information). To study the optical activities of Sr_18_Ge_9_O_5_S_31_, we also carried out the measurement of the solid‐state CD spectroscopy based on the sub‐millimeter sized crystals that were picked out from the solid‐state reactions. Because of the small crystal sizes and the similar morphologies, it is difficult to perfectly distinguish two enantiomers. But owing to the randomly unequal distribution of the enantiomers, the peak may also be observed in the odd numbers of selected crystals, i.e., exhibiting optical activity, which has been illustrated in KMgBO_3_.^[^ ^5b^ ^]^ Fortunately, a peak around 300 nm is also observed on the CD spectrum of Sr_18_Ge_9_O_5_S_31_ (Figure 4f), which is consistent with the corresponding ultraviolet‐visible absorption spectrum (Figure 4f). These suggest that Sr_18_Ge_9_O_5_S_31_ (*R*3) is optically active.

### 2.4 PFM Measurement

**Figure 5:**
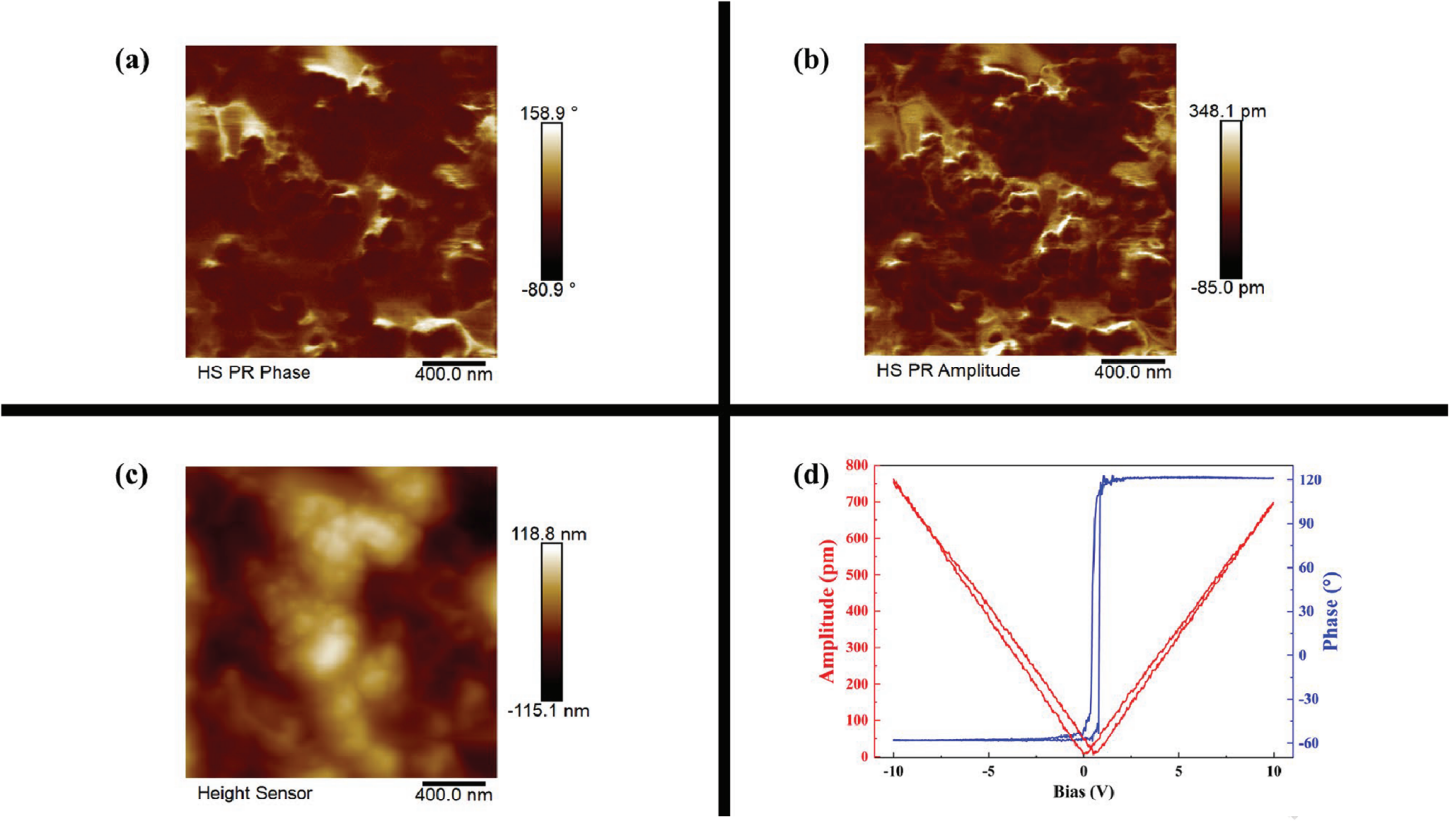
The piezoelectric force microscopy images of Sr_18_Ge_9_O_5_S_31_. a) Out‐of‐plane phase; b) out‐of‐plane amplitude; c) topography image; d) phase hysteresis loop and amplitude butterfly loop.

The *R*3 space group is a polar space group, thus, the ferroelectric, piezoelectric, and NLO properties of Sr_18_Ge_9_O_5_S_31_ were expected. As shown in **Figure** **5**a,b, the distinct phase and amplitude contrasts in different regions are observed in the PFM measurements, indicating the presence of diverse polarization directions. Furthermore, the appearance of the amplitude and phase images significantly deviates from that of the topographic image (Figure 5c), suggesting that their signals originate from domain structures rather than surface morphology. Remarkably, upon applying a 10 V flip bias, a phase‐reversal hysteresis loop is evident (Figure 5d), confirming the existence of 180° domain switching. Additionally, the presence of a characteristic butterfly loop pattern in variations of amplitude collectively substantiates the ferroelectric nature of Sr_18_Ge_9_O_5_S_31_. In order to ensure the reliability of PFM test results, the sample was measured by Sawer–Tower circuit method. The polarization versus applied electric field hysteresis curve is displayed in Figure S5 (Supporting Information), which is similar to ones observed in [Sr(DMF)‐(µ‐BDC)]_∞_ (DMF = *N,N*‐dimethylformamide; BDC^2−^ = benzene‐1,4‐dicarboxylate) and (DAMP)_3_(Cu_4_Br_4_)_2_(H_2_O)_3_ (DAMP = (S)−1,4‐diallyl‐2‐methylpiperazine).^[^ ^18^ ^]^ These indicate that Sr_18_Ge_9_O_5_S_31_ is ferroelectric.

### 2.5 NLO Properties

**Figure 6:**
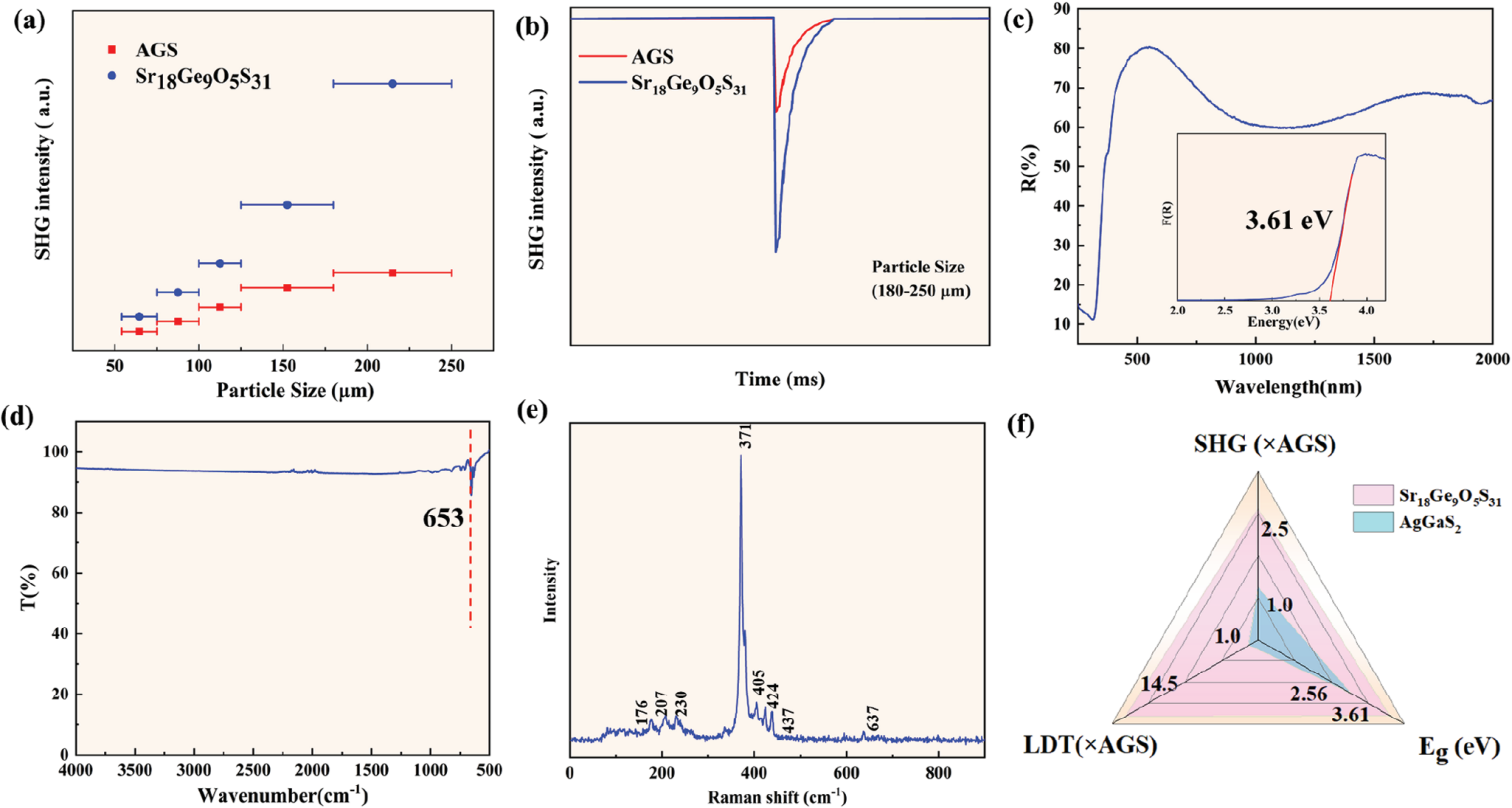
a) The SHG intensities versus particle sizes for Sr_18_Ge_9_O_5_S_31_ and AgGaS_2_ (AGS) as a reference; b) SHG intensities of Sr_18_Ge_9_O_5_S_31_ and AGS at a particle size of 180–250 µm; c) UV−vis−NIR diffuse reflectance spectrum measured with pure polycrystalline, bandgap (*E*_g_) (inserted), d) IR spectrum; e) Raman spectrum; f) Radar charts of the SHG response, *E*_g_, and laser damage threshold (LDT) of Sr_18_Ge_9_O_5_S_31_ and AGS.

The NLO properties of Sr_18_Ge_9_O_5_S_31_ were studied by the powder SHG measurement with the AGS as the reference by using a 2.09 µm laser based on the Kurtz–Perry technique.^[^ ^19^ ^]^ The curve of the SHG signal versus particle size, as shown in **Figure** **6**a, indicates that the SHG intensities increase with particle size, which suggests that the compound exhibits a phase‐matching (PM) nature. This PM nature facilitates the energy transfer from the fundamental light to the second harmonic and greatly enhances the conversion efficiency of the latter. Figure 6b shows that Sr_18_Ge_9_O_5_S_31_ displays a strong SHG response that is around 2.5 times that of AGS at the particle size of 180–250 µm. The NLO coefficients of Sr_18_Ge_9_O_5_S_31_ are comparable (or even larger) to those of other oxychalcogenides, such as SrZn_2_OS_2_ (2 × KH_2_PO_4_),^[^ ^20^ ^]^ Sr_5_Ga_8_O_3_S_14_ (0.8 × AGS),^[^ ^21^ ^]^ SrGeOS_2_ (0.4 × AGS),^[^ ^22^ ^]^ BaGeOS_2_ (0.5 × AGS),^[^ ^22^ ^]^ Sr_3_Ge_2_O_4_Se_3_ (0.8 × AGS),^[^ ^23^ ^]^ and Sr_3_[SnOSe_3_][CO_3_] (1.0 × AGS) (Table S4, Supporting Information).^[^ ^11^ ^]^

### 2.6 Optical Properties

In order to better indicate the importance of Sr_18_Ge_9_O_5_S_31_ as NLO crystal, more optical measurements are also carried out. The ultraviolet‐visible‐near infared diffuse reflectance spectrum of Sr_18_Ge_9_O_5_S_31_ is shown in Figure 6c and it was converted to the absorption based on the following Kubelka–Munk equation:^[^ ^24^ ^]^ $F\;(R)=\frac{K}{S}=\frac{{(1-R)}^{2}}{2R}$

where *R* is the reflectance, *K* is the absorption, and *S* is the scattering. On its absorption curve, elongating the linear part to zero, a large bandgap of 3.61 eV can be obtained for Sr_18_Ge_9_O_5_S_31_, which is higher than the bandgaps of commercially available IR NLO crystals such as AGS (2.70 eV),^[^ ^25^ ^]^ AgGaSe_2_ (1.80 eV),^[^ ^26^ ^]^ and ZnGeP_2_ (2.0 eV).^[^ ^27^ ^]^ Large bandgap is generally helpful for materials to produce a high laser demerge threshold (LDT). The LDT was measured using a Nd: YAG nanosecond laser (1064 nm, 1 Hz, 10 ns). The result demonstrates that Sr_18_Ge_9_O_5_S_31_ indeed has a high LDT (≈78.6 MW cm^−2^), which is more than 14.5 times that of AGS (≈5.4 MW cm^−2^).^[^ ^28^ ^]^ The IR and Raman spectra of Sr_18_Ge_9_O_5_S_31_ were analyzed, which show that Sr_18_Ge_9_O_5_S_31_ can exhibit a wide IR transmission without any significant absorption peaks to 653 cm^−1^ (≈15.3 µm, which can be attributed to the Ge─O stretching mode.) (Figure 6d,e).^[^ ^29^ ^]^ In addition, as shown in Figure 6e, the Raman spectrum shows that the characteristic peak 637 cm^−1^ was observed in the range of 4000–400 cm^−1^ belongs to the Ge─O stretching mode.^[^ ^29^ ^]^ The absorption peaks in the region from 176 to 437 cm^−1^ can be attributed to the vibration of the Ge─S bond.^[^ ^30^ ^]^ These also further confirm the existence of heteroanionic groups in the structure.

The experimental birefringence of Sr_18_Ge_9_O_5_S_31_ was measured using the cross‐polarizing microscope method with plate‐shaped crystals based on the formula *R* = Δ*n* × *d*, where *R*, Δ*n*, and *d* are retardation, birefringence, and thickness of the crystal, respectively.^[^ ^31^ ^]^ In the measurement, the observed interference color in cross‐polarized light was the third‐order green for Sr_18_Ge_9_O_5_S_31_ (Figure S6, Supporting Information). By comparing the Michal–Levy chart, the value of *R* is 1800 and *d* is 7.5 µm for Sr_18_Ge_9_O_5_S_31_. The orientation of such a utilized crystal was determined as [$\bar{1}$00] by using the “Index Crystal Faces” program in Bruker SMART APEX III (Figure S6, Supporting Information). Consequently, the measured birefringence is about 0.024.

### 2.7 The Relationship of Structure‐Property

The strong SHG response, large bandgap, and high LDT as well as the wide IR transmission imply that Sr_18_Ge_9_O_5_S_31_ would have the potential as an IR NLO crystal. Furthermore, the NLO properties of Sr_18_Ge_9_O_5_S_31_ were compared with the commercialized single‐anionic compound AGS (Figure 6f), it is clear that Sr_18_Ge_9_O_5_S_31_ can exhibit the better balance among the key properties, i.e., larger SHG response and higher bandgap than AGS. The excellent NLO properties of Sr_18_Ge_9_O_5_S_31_ can be attributed to its unique crystal structure. First, in Sr_18_Ge_9_O_5_S_31_, its BBUs, i.e., the hourglass‐like [Sr_3_OGeS_3_]^2+^ and [Sr_3_SGeS_3_]^2+^ groups, have a perfect aligned arrangement along *c* axis, which makes the microscopic second‐order susceptibility generated by the BBUs be able to better superposed. In order to better show the uniform arrangement of BBUs in the structure, the dipole moment calculations were also carried out based on a bond valance method.^[^ ^32^ ^]^ As shown in Figure S7 (Supporting Information), the net dipole moment from the GeOS_3_ and GeS_4_ tetrahedra points to the *c* direction, and a 8.24 Debye of net dipole moment is produced in a unit cell (Table S5, Supporting Information). The large net dipole moment and additive arrangements of BBUs are all favorable for Sr_18_Ge_9_O_5_S_31_ to generate a large SHG response. Meanwhile, compared to the single‐anion chalcogenides, introducing the large electronegative O atoms is also helpful for widening the bandgaps. That can be confirmed by the calculation of highest occupied molecular orbital‐lowest unoccupied molecular orbital (HOMO−LUMO) gaps of anion groups based on the Gaussian calculation. As shown in Figure S8, Supporting Information, it is clear that the heteroanionic [GeOS_3_] can produce larger HOMO−LUMO gap than single‐anionic ones, which is helpful for materials to generate wide bandgaps and high LDTs.

### 2.8 The First‐Principles Calculations

**Figure 7:**
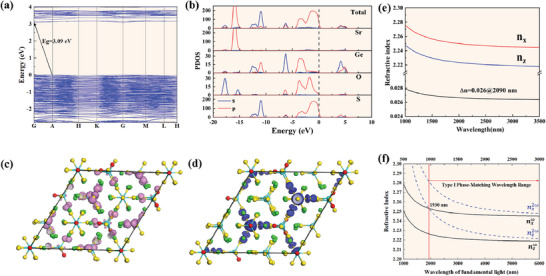
a) The band structure and b) density of states (DOS) diagram of Sr_18_Ge_9_O_5_S_31_; c) plots of the charge densities of the top of valence bands and d) the bottom of conduction bands; e) the calculated wavelength‐dependent birefringence Δn and f) calculated phase matching for Sr_18_Ge_9_O_5_S_31_ based on the first‐principles.

In order to better investigate the structure‐property relationship of Sr_18_Ge_9_O_5_S_31_, the first‐principles calculations using density functional theory (DFT) methods were also carried out.^[^ ^33^ ^]^ As illustrated in **Figure** **7**a, the band structure calculated based on the Perdew–Burke–Ernzerhof functional indicates an indirect bandgap of 3.09 eV for Sr_18_Ge_9_O_5_S_31_. However, the calculated value is smaller than the experimental result due to the commonly underestimated *E*_g_ by the DFT method. The partial density of states (PDOS) of Sr_18_Ge_9_O_5_S_31_ is displayed in Figure 7b, revealing that the valence band maximum (VBM) mainly consists of the O 2p, S 3p orbitals with a little Ge 4p orbital, and the conduction band minimum (CBM) is mainly dominated by the Ge 4s, 4p orbitals, with a little S 3p orbital, which can be distinguished more clearly in the plots of the charge densities of the VBM and the CBM of Sr_18_Ge_9_O_5_S_31_ in Figure 7c,d. These suggest that the [GeOS_3_] and [GeS_4_] tetrahedra play a major role in the optical properties of Sr_18_Ge_9_O_5_S_31_.

Furthermore, the second‐order NLO susceptibility of Sr_18_Ge_9_O_5_S_31_ was also calculated. It belongs to the point group of *C*_3_. Under the restriction of Kleinman's symmetry,^[^ ^34^ ^]^ it has four nonzero independent NLO susceptibility tensors, and the results show that *d*_11_ = 0.03 pm/V *d*_22_ = 8.76 pm/V, *d*_15_ = 9.54 pm/V, *d*_33_ = 13.64 pm/V, respectively, which are close to the experimental results. Based on its electronic structure, the birefringence of Sr_18_Ge_9_O_5_S_31_ was calculated, which is important for PM behavior. The calculated value of the static birefringence Δ*n* of Sr_18_Ge_9_O_5_S_31_ was 0.026@2090 nm, as depicted in Figure 7e. This is consistent with the commonly accepted moderate birefringence value and is favorable for achieving PM. In order to illustrate the relationship between birefringence and PM, we calculated the PM wavelength range based on the calculated birefringence (Figure 7f). The result shows that the Δ*n* value of Sr_18_Ge_9_O_5_S_31_ is sufficient to support PM.

## 3 Conclusion

In summary, a novel oxychalcogenide, Sr_18_Ge_9_O_5_S_31_ with chiral‐polar duality has been successfully designed and synthesized by symmetry‐breaking strategy. It crystallizes in one of the five point‐groups that possess both chiral and polar duality, namely the *C*_3_ point‐group. In this structure, the formation of hourglass‐like groups [Sr_3_OGeS_3_]^2+^ and [Sr_3_SGeS_3_]^2+^ breaks all the center of symmetry, twofold axis perpendicular to the principal axis of symmetry, mirror symmetry, and inversion axis symmetry, which facilitates the generation of duality (chirality and polarity). Two enantiomers of Sr_18_Ge_9_O_5_S_31_ have been identified by single crystal XRD. Its optical activity and ferroelectric properties were confirmed by CD spectroscopy and PFM, respectively. Furthermore, Sr_18_Ge_9_O_5_S_31_ exhibits excellent NLO properties, with a large SHG response (2.5 × AGS), wide bandgap (3.61 eV), and high LDT (14.5 × AGS) as well as the wide IR transmission (15.3 µm). These results demonstrate Sr_18_Ge_9_O_5_S_31_ may have the potential as an IR NLO crystal. Clearly, the polarity and chirality of Sr_18_Ge_9_O_5_S_31_ mainly originate from its hourglass‐like [Sr_3_OGeS_3_]^2+^ and [Sr_3_SGeS_3_]^2+^ groups. Therefore, constructing the hourglass‐like heteroanionic groups may be an effective strategy for the synthesis of materials with chiral‐polar duality.

## Conflict of Interest

The authors declare no conflict of interest.

## Supporting information

Supporting Information

## Data Availability

The data that support the findings of this study are available from the corresponding author upon reasonable request.
