# Supplementary material for: Chiral and Polar Duality Design of Heteroanionic Compounds: Sr18Ge9O5S31 Based on [Sr3OGeS3]2+ and [Sr3SGeS3]2+ Groups
Source: Adv Sci (Weinh). 2023 Dec 8;11(7):2306825. doi: 10.1002/advs.202306825 (PMC10870052; doi:10.1002/advs.202306825)
Supplement: Supplementary file 1 — Supporting Information [file ADVS-11-2306825-s001.pdf]

ADVANCED SCIENCE
Open Access

Supporting Information

for *Adv. Sci.*, DOI 10.1002/advs.202306825

Chiral and Polar Duality Design of Heteroanionic Compounds: $Sr_{18}Ge_9O_5S_{31}$ Based on $[Sr_3OGeS_3]^{2+}$ and $[Sr_3SGeS_3]^{2+}$ Groups

Shaoxin Cui, Hongping Wu, Xinkang Dong, Zhanggui Hu, Jiyang Wang, Yicheng Wu, Kenneth R. Poeppelmeier and Hongwei Yu**

Supporting Information
©Wiley-VCH 2021
69451 Weinheim, Germany

Chiral and Polar Duality Design of Heteroanionic Compounds: $Sr_{18}Ge_9O_5S_{31}$ Based on $[Sr_3OGeS_3]^{2+}$ and $[Sr_3SGeS_3]^{2+}$ Groups

Shaoxin Cui,[a] Hongping Wu,[a] Xinkang Dong,[a] Zhanggui Hu,[a] Jiyang Wang,[a] Yicheng Wu,[a] Kenneth R. Poeppelmeier*[b] and Hongwei Yu*[a]

Abstract: Chirality and polarity are two most important and representative symmetry-dependent properties. For polar structures, all the two-fold axes perpendicular to the principal axis of symmetry should be removed. For chiral structures, all the mirror-related symmetries and inversion axes should be removed. Especially for duality (polarity and chirality), all of the above symmetries should be broken and that also represents the highest-level challenge. Herein, we proposed a new symmetry-breaking strategy, *i.e.,* employing heteroanionic groups to construct hourglass-like $[Sr_3OGeS_3]^{2+}$ and $[Sr_3SGeS_3]^{2+}$ groups, to design and synthesize a new oxychalcogenide, $Sr_{18}Ge_9O_5S_{31}$ with chiral-polar duality. The presence of two enantiomers of $Sr_{18}Ge_9O_5S_{31}$ is confirmed by the single-crystal X-ray diffraction. Its optical activity and ferroelectricity are also studied by the solid-state circular dichroism (CD) spectroscopy and piezoresponse force microscopy (PFM), respectively. Further property measurements show that $Sr_{18}Ge_9O_5S_{31}$ possesses excellent nonlinear optical (NLO) properties, including the strong second harmonic generation (SHG) efficiency (~2.5 × AGS), large band gap (3.61 eV), and wide mid-infrared transparent region (~15.3 μm). These indicate that the unique microstructure groups of heteroanionic materials are conducive to realize the symmetry-breaking and able to provide some inspiration for exploring the chiral-polar duality materials.

DOI: 10.1002/anie.2021XXXXX

Table of Contents

Experimental Section......3
Table S1 **(Crystal data and structure refinement for $Sr_{18}Ge_9O_5S_{31}$)**......5
Table S2 **(Selected bond distances and angles)**......6
Table S3 **(Atomic coordinates, displacement parameters and BVS)**......14
Table S4 **(Property comparison of $Sr_{18}Ge_9O_5S_{31}$ with other reported oxychalcogenides)**......16
Table S5 **(The dipole moment calculations)**......17
Figure S1 **(The experimental and calculated XRD patterns)**......18
Figure S2 **(The coordination environment of Sr atoms)**......19
Figure S3 **(The morphology of enantiomer 1, enantiomer 2, and polycrystalline for $Sr_{18}Ge_9O_5S_{31}$)**......20

Figure S4 **(The theoretical morphology of enantiomer 1 and enantiomer 2)**....21
Figure S5 **(The polarization versus applied electric field (*P-E*) hysteresis curve of $Sr_{18}Ge_9O_5S_{31}$)**....22
Figure S6 **(Crystal thickness picture of $Sr_{18}Ge_9O_5S_{31}$)**....23
Figure S7 **(Schematic drawing of the direction and magnitude of the dipole moments)**....24
Figure S8 **(The HOMO−LUMO gaps of Ge(3)OS_3, Ge(4)OS_3, and GaS_4)**....25
References....26

Experimental Procedures

Single Crystal Synthesis. All staring materials, SrS (99.99%), Ge (99.99%), S (99.999%), and GeO_2 (99.9%) were directly purchased from Aladdin Co., Ltd. Without further purification. All manipulations are carried out in an Ar-filled glove box. Single crystal of $Sr_{18}Ge_9O_5S_{31}$ was synthesized using 18:2.5:6.5:13 ratios of SrS, GeO_2, Ge and S with a total mass of 500 mg as the starting materials. After being ground to fine powder, the mixture was loaded into quartz tube and further evacuated to 1×10^{-3} Pa, and then sealed by flame. The tube was placed into a muffle furnace, heated from 30 °C to 950 °C at a rate of 150 °C/h and hold at this temperature for 24 h, gradually cooled to 700 °C at a speed of 5 °C/h, and finally the furnace was turned off. The single crystal of $Sr_{18}Ge_9O_5S_{31}$ was obtained.

Structure Determination. The powder X-ray diffraction (PXRD) pattern was collected setting from the 2θ range 10-70° with a step width size of 0.01° and a step time of 2s on an automated SmartLab 3KW powder X-ray diffractometer using Cu-K_α radiation (λ = 1.54057 Å) radiation. The purity of compound $Sr_{18}Ge_9O_5S_{31}$ was verified by PXRD. The crystal structure of $Sr_{18}Ge_9O_5S_{31}$ was determined by single-crystal XRD on a Bruker SMART APEX III CCD diffractometer using Mo-K_α radiation (λ = 0.71073 Å) at 293(2) K and the data was integrated with the SAINT program.[1] All calculations were implemented with programs from the SHELXTL crystallographic software package.[2] Its crystal structure was solved by direct methods using SHELXS and refined with full-matrix least-squares methods on F^2 with anisotropic thermal parameters for all atoms.[3] Crystallographic data for the structures reported in this paper have been deposited with the Cambridge Crystallographic Data Centre. CCDC 2280602-2280603 contain the supplementary crystallographic data for this paper. These data can be obtained free of charge from The Cambridge Crystallographic Data Centre via www.ccdc.cam.ac.uk/data_request/cif. Crystal data and structure refinement parameters were given in Table S1, Supporting Information. Some structural parameters including interatomic distances and angles, final refined atomic positions and isotropic thermal parameters are listed in Table S2 and Table S3, Supporting Information, respectively.

Solid-state Circular Dichroism spectrum. The measurement of CD spectrum was performed under ambient conditions on a JASCO J-1500 CD spectrometer. The sample for CD measurement was prepared though pressing the $Sr_{18}Ge_9O_5S_{31}$ powder and KBr powder ($Sr_{18}Ge_9O_5S_{31}$: KBr=1:150) into a wafer.

***Scanning Electron Microscopy*:** The morphologies of the crystals were measured on a field-emission scanning electron microscope (Quanta FEG 250) made by FEI.

PFM characterization. The PFM measurement was carried out on a commercial piezoresponse force microscope (Bruker Dimension Icon) with the high-voltage package and in-situ heating stage. PFM is based on the atomic force microscopy (AFM), with an AC drive voltage applied to the conductive tip. Conductive Pt/Ir-coated silicon probes (EFM, Nanoworld) were used for domain imaging and polarization switching studies, with a nominal spring constant of ~2.8 nN/nm and a free-air resonance frequency of ~75 kHz. Since the amplitude of the low-frequency vertical PFM within the noise level of the quadrant photodetector of the AFM, we performed the PFM experiments at contact resonance.

Ferroelectric Measurements. The P−E hysteresis loop was measured with the standard Sawyer−Tower method (Premier II, Radiant, USA).

UV−Vis−NIR Diffuse reflectance. The UV−vis−NIR diffuse reflectance spectrum of $Sr_{18}Ge_9O_5S_{31}$ in the range of 250−2000 nm was measured on Shimadzu SolidSpec-3700DUV with $BaSO_4$ as a reference. The band gap was estimated on basis of the absorption spectrum that was derived from the reflection spectrum using the Kubelka-Munk formula.[4]

IR spectroscopy and Raman spectroscopy. The IR spectrum in the range of 4000−400 cm^{-1} was recorded on a Fourier transform IR spectrometer using Nicolet iS50FT. The Raman spectrum was collected with powder of $Sr_{18}Ge_9O_5S_{31}$ on a WITEC alpha300R spectrometer equipped with a CCD detector using 532 nm radiations from a diode laser. The measurement range is 90-9000 cm^{-1}. The laser spot diameter is shorter than 1 μm and the test time is less than 60s. As shown in Figure 6e, the characteristic peak 637 cm^{-1} were observed in the range of 4000-400 cm^{-1}, which belong to the Ge–O stretching mode.[5] The absorption peaks in the region from 176 to 437 cm^{-1} can be attributed to the vibration of the Ge-S bond.[6]

Second harmonic generation measurement. The SHG signals of $Sr_{18}Ge_9O_5S_{31}$ and benchmark AGS were investigated under incident laser radiation of 2090 nm by the modified Kurtz-Perry method, respectively.[7] Samples $Sr_{18}Ge_9O_5S_{31}$ and AGS were sieved into several distinct particle size ranges (54−75, 75−100, 100−125, 125−180, and 180−250 μm) for the PM measurements. The SHG signals were detected by a charge-coupled device. The AGS samples with similar particle size ranges were served as the standard samples.

Laser damage threshold measurement. The LDT of the $Sr_{18}Ge_9O_5S_{31}$ and AGS powder at the particle size range of 180−250 μm were evaluated using a high-power laser irradiation of 1064 nm (pulse width τ_p = 10 ns) by the single-pulse method.[8] The measurement

processes were performed by gradually increasing the laser power until the damaged spot was observed under a microscope. The damage thresholds were derived from the equation $I_{(threshold)} = EI(\pi r^2\ \tau_p)$, where E is the laser energy of a single pulse, r is the spot radius, and τ_p is the pulse width.

Computational Methods. The Gaussian 09 package was employed to explore the electronic structures of anionic groups at molecular level. DFT method at the B3LYP level with 6-31G basis sets was performed to calculate the cluster. The electronic band structures, the partial density of states and optical properties for $Sr_{18}Ge_9O_5S_{31}$ were carried out using the CASTEP package based on density functional theory (DFT).[9] Generalized gradient approximation (GGA) parametrized by Perdew-Burke-Ernzerhof (PBE) functional was chosen for the exchange-correlation energy, and the pseudopotential was set as norm-conserving pseudopotential (NCP).[10] The valence electrons were set as: Sr: $4s^24p^65s^2$, Ge: $4s^24p^2$, O: $2s^22p^4$, and S: $3s^23p^4$. The plane wave energy cutoff value was set at 810.0 eV. The numerical integration of the Brillouin zones was performed using 2 × 2 × 2 Monkhorst-Pack k-point meshes.[11] For calculating the optical properties, the same Monkhorst-pack grid and plane-wave cutoff energy were used and the dielectric function is defined as $\varepsilon(\omega) = \varepsilon_1(\omega) + i\varepsilon_2(\omega)$,[3, 9] in which real part $\varepsilon_1(\omega)$, refractive index n(ω) were obtained by the Kramers-Kroning transform.[12] The optical property calculations was scissor corrected (0.52 eV for $Sr_{18}Ge_9O_5S_{31}$) by the energy gap difference between the PBE and the experimental values. As important parameters for NLO crystals, SHG coefficients were also calculated.

The SHG coefficients calculations are performed based on the band wave functions by using the so-called length-gauge formalism derived by Aversa and Sipe[13] at a zero frequency limit. The static second-order nonlinear susceptibilities $\chi^{(2)}_{\alpha\beta\gamma}$ can be simplified as,[14]

$$\chi^{(2)}_{\alpha\beta\gamma}=\chi^{(2)}_{\alpha\beta\gamma}(VE)+\chi^{(2)}_{\alpha\beta\gamma}(VH)+\chi^{(2)}_{\alpha\beta\gamma}(\text{two-bands})$$

In this sum-over-states type formalism, the total SHG coefficient $\chi^{(2)}$ is divided into contributions from Virtual-Hole (VH), Virtual-Electron (VE) and Two-Band (TB) processes. Based on this, we calculated the origin of the SHG coefficient.

Table S1. Crystal data and structure refinement for $Sr_{18}Ge_9O_5S_{31}$.

Empirical formula	enantiomer 1	enantiomer 2
Formula weight	3304.3	3304.3
Temperature (K)	293(2)	273(2)
Crystal system	Trigonal	Trigonal
Space group	*R*3	*R*3
Z	3	3
a (Å)	16.9427(5)	16.9443(7)
c (Å)	18.0713(14)	18.0737(11)
D_c (g cm -3)	3.664	3.663
μ (mm -1)	21.414	21.407
F (000)	4524	4524
Radiation	Mo-K_α (λ = 0.71073)	Mo-K_α (λ = 0.71073)
2θ range (°)	2.404 to 25.316°	2.404 to 23.811°
Reflections collected	10740	12320
Independent reflections/R_{int}	3609/0.0710	3069/0.0981
GOOF on F^2	1.030	1.021
R_1[a)], wR_2 (I >2σ(I)) [b)]	0.0470, 0.0861	0.0528, 0.1069
R_1, wR_2 (all data)	0.0632, 0.0929	0.0763, 0.1174

largest diff. peak and hole (e·Å^{-3})	1.237, -1.282	2.124, -1.206
Flack parameter	0.090(18)	0.061(17)

[a]$R_1 = \Sigma||F_o| — |F_c||/\Sigma|F_o|$, [b]$wR_2 = \Sigma w(F_o^2 — F_c^2)^2/\Sigma w(F_o^2)^2]^{1/2}$

Table S2a. Selected bond distances (Å) and angles (degrees) for $Sr_{18}Ge_9O_5S_{31}$ (enantiomer 1).

Sr(1)-O(3)	2.488(6)	Sr(5)-S(6)#12	3.109(5)
Sr(1)-S(1)	2.990(5)	Sr(5)-S(10)	3.129(5)
Sr(1)-S(8)	3.025(6)	Sr(5)-S(4)#6	3.177(6)
Sr(1)-S(2)	3.050(5)	Sr(5)-S(2)#13	3.199(6)
Sr(1)-S(9)	3.126(6)	Sr(6)-S(5)#10	2.901(5)
Sr(1)-S(6)#1	3.198(5)	Sr(6)-S(3)	2.988(5)
Sr(1)-S(6)	3.235(5)	Sr(6)-S(2)	2.989(6)
Sr(2)-O(1)#4	2.422(8)	Sr(6)-S(6)#10	3.063(5)
Sr(2)-S(1)	2.986(5)	Sr(6)-S(7)#1	3.094(5)
Sr(2)-S(4)	3.026(5)	Sr(6)-S(8)	3.163(5)
Sr(2)-S(10)#5	3.119(6)	Sr(6)-S(1)#10	3.196(5)
Sr(2)-S(10)	3.153(6)	Sr(6)-S(9)#10	3.468(5)
Sr(2)-S(5)#5	3.168(5)	Ge(1)-S(4)	2.186(5)
Sr(2)-S(8)#2	3.250(6)	Ge(1)-S(1)	2.206(5)
Sr(2)-S(5)	3.295(5)	Ge(1)-S(2)	2.218(5)
Sr(3)-O(2)#1	2.466(12)	Ge(1)-S(3)	2.220(5)
Sr(3)-S(11)#7	2.975(5)	Ge(2)-O(2)	1.777(12)
Sr(3)-S(7)#1	3.091(5)	Ge(2)-S(7)	2.208(5)
Sr(3)-S(9)#8	3.099(5)	Ge(2)-S(5)	2.213(5)
Sr(3)-S(4)#9	3.120(5)	Ge(2)-S(6)	2.227(5)
Sr(3)-S(9)#10	3.162(6)	Ge(3)-O(1)	1.79(2)
Sr(3)-S(2)	3.168(5)	Ge(3)-S(8)#3	2.215(5)
Sr(4)-O(2)#12	2.631(12)	Ge(3)-S(8)	2.215(5)
Sr(4)-S(5)#12	3.041(5)	Ge(3)-S(8)#1	2.215(5)
Sr(4)-S(7)	3.060(5)	Ge(4)-O(3)	1.77(2)
Sr(4)-S(3)	3.095(5)	Ge(4)-S(9)#3	2.207(5)
Sr(4)-S(4)#10	3.203(6)	Ge(4)-S(9)#1	2.207(5)
Sr(4)-S(10)	3.217(5)	Ge(4)-S(9)	2.207(5)
Sr(4)-S(1)	3.230(5)	Ge(5)-S(11)	2.192(10)
Sr(4)-S(8)	3.368(5)	Ge(5)-S(10)#5	2.208(5)
Sr(5)-O(2)#12	2.477(12)	Ge(5)-S(10)#6	2.208(5)

Sr(5)-S(3)#6	2.995(5)	Ge(5)-S(10)	2.208(5)
Sr(5)-S(7)	3.020(5)		
O(3)-Sr(1)-S(1)	141.78(12)	S(4)#10-Sr(4)-S(1)	141.92(13)
O(3)-Sr(1)-S(8)	99.5(5)	S(10)-Sr(4)-S(1)	78.20(14)
S(1)-Sr(1)-S(8)	81.03(14)	O(2)#12-Sr(4)-S(8)	137.0(3)
O(3)-Sr(1)-S(2)	145.04(12)	S(5)#12-Sr(4)-S(8)	69.96(13)
S(1)-Sr(1)-S(2)	73.16(13)	S(7)-Sr(4)-S(8)	100.41(14)
S(8)-Sr(1)-S(2)	85.14(14)	S(3)-Sr(4)-S(8)	70.41(13)
O(3)-Sr(1)-S(9)	69.5(5)	S(4)#10-Sr(4)-S(8)	92.95(14)
S(1)-Sr(1)-S(9)	104.30(14)	S(10)-Sr(4)-S(8)	150.80(15)
S(8)-Sr(1)-S(9)	167.81(14)	S(1)-Sr(4)-S(8)	72.61(13)
S(2)-Sr(1)-S(9)	106.83(15)	O(2)#12-Sr(5)-S(3)#6	140.7(3)
O(3)-Sr(1)-S(6)#1	74.21(15)	O(2)#12-Sr(5)-S(7)	76.6(3)
S(1)-Sr(1)-S(6)#1	141.90(14)	S(3)#6-Sr(5)-S(7)	142.64(16)
S(8)-Sr(1)-S(6)#1	109.93(14)	O(2)#12-Sr(5)-S(6)#12	68.4(3)
S(2)-Sr(1)-S(6)#1	71.69(13)	S(3)#6-Sr(5)-S(6)#12	116.46(14)
S(9)-Sr(1)-S(6)#1	72.79(13)	S(7)-Sr(5)-S(6)#12	73.62(14)
O(3)-Sr(1)-S(6)	73.51(15)	O(2)#12-Sr(5)-S(10)	73.8(3)
S(1)-Sr(1)-S(6)	69.09(13)	S(3)#6-Sr(5)-S(10)	106.51(14)
S(8)-Sr(1)-S(6)	101.95(14)	S(7)-Sr(5)-S(10)	77.12(14)
S(2)-Sr(1)-S(6)	139.79(14)	S(6)#12-Sr(5)-S(10)	136.34(14)
S(9)-Sr(1)-S(6)	70.54(13)	O(2)#12-Sr(5)-S(4)#6	145.1(3)
S(6)#1-Sr(1)-S(6)	137.56(19)	S(3)#6-Sr(5)-S(4)#6	70.30(14)
O(1)#4-Sr(2)-S(1)	139.10(18)	S(7)-Sr(5)-S(4)#6	73.54(14)
O(1)#4-Sr(2)-S(4)	143.16(19)	S(6)#12-Sr(5)-S(4)#6	118.53(14)
S(1)-Sr(2)-S(4)	73.82(13)	S(10)-Sr(5)-S(4)#6	82.15(14)
O(1)#4-Sr(2)-S(10)#5	84.4(4)	O(2)#12-Sr(5)-S(2)#13	71.8(3)
S(1)-Sr(2)-S(10)#5	125.03(15)	S(3)#6-Sr(5)-S(2)#13	73.64(14)
S(4)-Sr(2)-S(10)#5	84.80(14)	S(7)-Sr(5)-S(2)#13	139.06(15)
O(1)#4-Sr(2)-S(10)	83.7(4)	S(6)#12-Sr(5)-S(2)#13	70.94(13)
S(1)-Sr(2)-S(10)	82.91(13)	S(10)-Sr(5)-S(2)#13	116.85(14)
S(4)-Sr(2)-S(10)	124.03(15)	S(4)#6-Sr(5)-S(2)#13	142.89(14)
S(10)#5-Sr(2)-S(10)	68.28(18)	S(5)#10-Sr(6)-S(3)	130.11(15)
O(1)#4-Sr(2)-S(5)#5	75.41(17)	S(5)#10-Sr(6)-S(2)	138.56(16)
S(1)-Sr(2)-S(5)#5	136.68(14)	S(3)-Sr(6)-S(2)	76.73(15)
S(4)-Sr(2)-S(5)#5	67.77(13)	S(5)#10-Sr(6)-S(6)#10	75.92(14)
S(10)#5-Sr(2)-S(5)#5	71.08(13)	S(3)-Sr(6)-S(6)#10	119.91(14)
S(10)-Sr(2)-S(5)#5	135.65(13)	S(2)-Sr(6)-S(6)#10	121.84(14)

O(1)#4-Sr(2)-S(8)#2	66.6(5)	S(5)#10-Sr(6)-S(7)#1	78.52(14)
S(1)-Sr(2)-S(8)#2	97.56(14)	S(3)-Sr(6)-S(7)#1	149.21(16)
S(4)-Sr(2)-S(8)#2	98.78(14)	S(2)-Sr(6)-S(7)#1	73.03(14)
S(10)#5-Sr(2)-S(8)#2	136.03(14)	S(6)#10-Sr(6)-S(7)#1	73.24(14)
S(10)-Sr(2)-S(8)#2	134.84(14)	S(5)#10-Sr(6)-S(8)	76.51(14)
S(5)#5-Sr(2)-S(8)#2	70.06(13)	S(3)-Sr(6)-S(8)	74.67(13)
O(1)#4-Sr(2)-S(5)	72.93(16)	S(2)-Sr(6)-S(8)	83.79(14)
S(1)-Sr(2)-S(5)	66.19(13)	S(6)#10-Sr(6)-S(8)	151.70(15)
S(4)-Sr(2)-S(5)	136.03(13)	S(7)#1-Sr(6)-S(8)	107.22(15)
S(10)#5-Sr(2)-S(5)	133.28(14)	S(5)#10-Sr(6)-S(1)#10	68.49(14)
S(10)-Sr(2)-S(5)	69.01(13)	S(3)-Sr(6)-S(1)#10	74.54(14)
S(5)#5-Sr(2)-S(5)	136.31(19)	S(2)-Sr(6)-S(1)#10	150.45(14)
S(8)#2-Sr(2)-S(5)	70.12(13)	S(6)#10-Sr(6)-S(1)#10	68.76(13)
O(2)#1-Sr(3)-S(11)#7	103.5(3)	S(7)#1-Sr(6)-S(1)#10	134.29(14)
O(2)#1-Sr(3)-S(7)#1	70.1(3)	S(8)-Sr(6)-S(1)#10	95.15(15)
S(11)#7-Sr(3)-S(7)#1	172.75(18)	S(5)#10-Sr(6)-S(9)#10	143.63(15)
O(2)#1-Sr(3)-S(9)#8	145.0(3)	S(3)-Sr(6)-S(9)#10	68.00(12)
S(11)#7-Sr(3)-S(9)#8	81.57(16)	S(2)-Sr(6)-S(9)#10	69.89(13)
S(7)#1-Sr(3)-S(9)#8	105.62(14)	S(6)#10-Sr(6)-S(9)#10	68.14(12)
O(2)#1-Sr(3)-S(4)#9	70.2(3)	S(7)#1-Sr(6)-S(9)#10	95.87(14)
S(11)#7-Sr(3)-S(4)#9	108.14(12)	S(8)-Sr(6)-S(9)#10	138.06(13)
S(7)#1-Sr(3)-S(4)#9	73.40(14)	S(1)#10-Sr(6)-S(9)#10	92.81(14)
S(9)#8-Sr(3)-S(4)#9	75.28(13)	S(4)-Ge(1)-S(1)	110.59(19)
O(2)#1-Sr(3)-S(9)#10	144.1(3)	S(4)-Ge(1)-S(2)	113.2(2)
S(11)#7-Sr(3)-S(9)#10	80.53(16)	S(1)-Ge(1)-S(2)	108.93(19)
S(7)#1-Sr(3)-S(9)#10	102.55(14)	S(4)-Ge(1)-S(3)	107.7(2)
S(9)#8-Sr(3)-S(9)#10	70.70(18)	S(1)-Ge(1)-S(3)	102.44(19)
S(4)#9-Sr(3)-S(9)#10	143.22(16)	S(2)-Ge(1)-S(3)	113.5(2)
O(2)#1-Sr(3)-S(2)	72.5(3)	O(2)-Ge(2)-S(7)	107.9(4)
S(11)#7-Sr(3)-S(2)	104.47(12)	O(2)-Ge(2)-S(5)	104.1(4)
S(7)#1-Sr(3)-S(2)	70.67(13)	S(7)-Ge(2)-S(5)	115.4(2)
S(9)#8-Sr(3)-S(2)	140.55(15)	O(2)-Ge(2)-S(6)	104.8(4)
S(4)#9-Sr(3)-S(2)	134.91(14)	S(7)-Ge(2)-S(6)	112.1(2)
S(9)#10-Sr(3)-S(2)	71.97(13)	S(5)-Ge(2)-S(6)	111.5(2)
O(2)#12-Sr(4)-S(5)#12	67.2(3)	O(1)-Ge(3)-S(8)#3	104.97(17)
O(2)#12-Sr(4)-S(7)	73.8(3)	O(1)-Ge(3)-S(8)	104.97(17)
S(5)#12-Sr(4)-S(7)	76.96(14)	S(8)#3-Ge(3)-S(8)	113.57(14)
O(2)#12-Sr(4)-S(3)	133.0(3)	O(1)-Ge(3)-S(8)#1	104.97(17)

S(5)#12-Sr(4)-S(3)	123.08(14)	S(8)#3-Ge(3)-S(8)#1	113.57(14)
S(7)-Sr(4)-S(3)	149.52(15)	S(8)-Ge(3)-S(8)#1	113.57(14)
O(2)#12-Sr(4)-S(4)#10	67.1(3)	O(3)-Ge(4)-S(9)#3	108.61(17)
S(5)#12-Sr(4)-S(4)#10	67.14(13)	O(3)-Ge(4)-S(9)#1	108.61(17)
S(7)-Sr(4)-S(4)#10	134.42(14)	S(9)#3-Ge(4)-S(9)#1	110.32(16)
S(3)-Sr(4)-S(4)#10	75.93(13)	O(3)-Ge(4)-S(9)	108.61(17)
O(2)#12-Sr(4)-S(10)	70.4(3)	S(9)#3-Ge(4)-S(9)	110.32(16)
S(5)#12-Sr(4)-S(10)	134.18(14)	S(9)#1-Ge(4)-S(9)	110.32(16)
S(7)-Sr(4)-S(10)	75.25(13)	S(11)-Ge(5)-S(10)#5	113.05(17)
S(3)-Sr(4)-S(10)	98.43(14)	S(11)-Ge(5)-S(10)#6	113.05(17)
S(4)#10-Sr(4)-S(10)	110.86(14)	S(10)#5-Ge(5)-S(10)#6	105.67(19)
O(2)#12-Sr(4)-S(1)	144.9(3)	S(11)-Ge(5)-S(10)	113.05(17)
S(5)#12-Sr(4)-S(1)	133.32(15)	S(10)#5-Ge(5)-S(10)	105.67(19)
S(7)-Sr(4)-S(1)	83.46(13)	S(10)#6-Ge(5)-S(10)	105.66(19)
S(3)-Sr(4)-S(1)	66.07(13)		

Symmetry transformations used to generate equivalent atoms:
#1 -y,x-y,z #2 -y-1/3,x-y+1/3,z+1/3 #3 -x+y,-x,z
#4 x-1/3,y+1/3,z+1/3 #5 -x+y-1,-x,z #6 -y,x-y+1,z
#7 x-1/3,y-2/3,z+1/3 #8 -y-2/3,x-y-1/3,z-1/3 #9 -x+y-1,-x-1,z
#10 -x+y-2/3,-x-1/3,z-1/3 #11 x-2/3,y-1/3,z-1/3 #12 -y+1/3,x-y+2/3,z-1/3
#13 x+1/3,y+2/3,z-1/3 #14 -x+y-1/3,-x+1/3,z+1/3 #15 x+1/3,y-1/3,z-1/3
#16 x+2/3,y+1/3,z+1/3 #17 -x+y-1/3,-x-2/3,z+1/3 #18 -y-1,x-y,z
#19 -y-2/3,x-y+2/3,z-1/3

Table S2b. Selected bond distances (Å) and angles (degrees) for $Sr_{18}Ge_9O_5S_{31}$ (enantiomer 2).

Sr(1)-O(3)	2.472(7)	Sr(5)-S(6)#6	3.098(8)
Sr(1)-S(1)	2.991(7)	Sr(5)-S(10)#6	3.136(8)
Sr(1)-S(8)	3.008(8)	Sr(5)-S(4)	3.164(8)
Sr(1)-S(2)	3.041(7)	Sr(5)-S(2)#14	3.211(8)
Sr(1)-S(9)#1	3.135(8)	Sr(6)-S(5)	2.898(8)
Sr(1)-S(6)#2	3.196(8)	Sr(6)-S(3)	2.985(8)
Sr(1)-S(6)#3	3.242(8)	Sr(6)-S(2)	2.986(8)
Sr(2)-O(1)#5	2.429(11)	Sr(6)-S(6)	3.053(8)
Sr(2)-S(1)	2.982(7)	Sr(6)-S(7)#2	3.096(8)
Sr(2)-S(4)	3.024(7)	Sr(6)-S(8)	3.175(8)
Sr(2)-S(10)#6	3.126(8)	Sr(6)-S(1)#14	3.194(8)
Sr(2)-S(10)#4	3.155(8)	Sr(6)-S(9)#15	3.460(7)

Sr(2)-S(5)#5	3.176(8)	Ge(1)-S(4)	2.195(7)
Sr(2)-S(8)#3	3.259(8)	Ge(1)-S(1)	2.207(8)
Sr(2)-S(5)#3	3.303(8)	Ge(1)-S(2)	2.211(7)
Sr(3)-O(2)	2.440(17)	Ge(1)-S(3)	2.219(8)
Sr(3)-S(11)	2.981(7)	Ge(2)-O(2)	1.787(16)
Sr(3)-S(9)#9	3.097(7)	Ge(2)-S(7)	2.206(8)
Sr(3)-S(7)	3.108(8)	Ge(2)-S(5)	2.212(8)
Sr(3)-S(4)#10	3.121(8)	Ge(2)-S(6)	2.238(8)
Sr(3)-S(9)#11	3.167(8)	Ge(3)-O(1)	1.78(3)
Sr(3)-S(2)#12	3.179(7)	Ge(3)-S(8)#4	2.220(7)
Sr(4)-O(2)	2.627(16)	Ge(3)-S(8)#1	2.220(7)
Sr(4)-S(5)	3.030(8)	Ge(3)-S(8)	2.220(7)
Sr(4)-S(7)#2	3.040(8)	Ge(4)-O(3)	1.84(3)
Sr(4)-S(3)#1	3.098(7)	Ge(4)-S(9)#1	2.215(7)
Sr(4)-S(4)#10	3.208(8)	Ge(4)-S(9)#4	2.215(7)
Sr(4)-S(10)	3.213(8)	Ge(4)-S(9)	2.215(7)
Sr(4)-S(1)#1	3.229(8)	Ge(5)-S(11)	2.182(14)
Sr(4)-S(8)#1	3.373(8)	Ge(5)-S(10)#16	2.208(7)
Sr(5)-O(2)#6	2.499(16)	Ge(5)-S(10)#17	2.208(7)
Sr(5)-S(3)	2.999(7)	Ge(5)-S(10)	2.208(7)
Sr(5)-S(7)#5	3.026(8)		
O(3)-Sr(1)-S(1)	141.78(12)	S(4)#10-Sr(4)-S(1)	141.92(13)
O(3)-Sr(1)-S(8)	99.5(5)	S(10)-Sr(4)-S(1)	78.20(14)
S(1)-Sr(1)-S(8)	81.03(14)	O(2)#12-Sr(4)-S(8)	137.0(3)
O(3)-Sr(1)-S(2)	145.04(12)	S(5)#12-Sr(4)-S(8)	69.96(13)
S(1)-Sr(1)-S(2)	73.16(13)	S(7)-Sr(4)-S(8)	100.41(14)
S(8)-Sr(1)-S(2)	85.14(14)	S(3)-Sr(4)-S(8)	70.41(13)
O(3)-Sr(1)-S(9)	69.5(5)	S(4)#10-Sr(4)-S(8)	92.95(14)
S(1)-Sr(1)-S(9)	104.30(14)	S(10)-Sr(4)-S(8)	150.80(15)
S(8)-Sr(1)-S(9)	167.81(14)	S(1)-Sr(4)-S(8)	72.61(13)
S(2)-Sr(1)-S(9)	106.83(15)	O(2)#12-Sr(5)-S(3)#6	140.7(3)
O(3)-Sr(1)-S(6)#1	74.21(15)	O(2)#12-Sr(5)-S(7)	76.6(3)
S(1)-Sr(1)-S(6)#1	141.90(14)	S(3)#6-Sr(5)-S(7)	142.64(16)
S(8)-Sr(1)-S(6)#1	109.93(14)	O(2)#12-Sr(5)-S(6)#12	68.4(3)
S(2)-Sr(1)-S(6)#1	71.69(13)	S(3)#6-Sr(5)-S(6)#12	116.46(14)
S(9)-Sr(1)-S(6)#1	72.79(13)	S(7)-Sr(5)-S(6)#12	73.62(14)
O(3)-Sr(1)-S(6)	73.51(15)	O(2)#12-Sr(5)-S(10)	73.8(3)
S(1)-Sr(1)-S(6)	69.09(13)	S(3)#6-Sr(5)-S(10)	106.51(14)

S(8)-Sr(1)-S(6)	101.95(14)	S(7)-Sr(5)-S(10)	77.12(14)
S(2)-Sr(1)-S(6)	139.79(14)	S(6)#12-Sr(5)-S(10)	136.34(14)
S(9)-Sr(1)-S(6)	70.54(13)	O(2)#12-Sr(5)-S(4)#6	145.1(3)
S(6)#1-Sr(1)-S(6)	137.56(19)	S(3)#6-Sr(5)-S(4)#6	70.30(14)
O(1)#4-Sr(2)-S(1)	139.10(18)	S(7)-Sr(5)-S(4)#6	73.54(14)
O(1)#4-Sr(2)-S(4)	143.16(19)	S(6)#12-Sr(5)-S(4)#6	118.53(14)
S(1)-Sr(2)-S(4)	73.82(13)	S(10)-Sr(5)-S(4)#6	82.15(14)
O(1)#4-Sr(2)-S(10)#5	84.4(4)	O(2)#12-Sr(5)-S(2)#13	71.8(3)
S(1)-Sr(2)-S(10)#5	125.03(15)	S(3)#6-Sr(5)-S(2)#13	73.64(14)
S(4)-Sr(2)-S(10)#5	84.80(14)	S(7)-Sr(5)-S(2)#13	139.06(15)
O(1)#4-Sr(2)-S(10)	83.7(4)	S(6)#12-Sr(5)-S(2)#13	70.94(13)
S(1)-Sr(2)-S(10)	82.91(13)	S(10)-Sr(5)-S(2)#13	116.85(14)
S(4)-Sr(2)-S(10)	124.03(15)	S(4)#6-Sr(5)-S(2)#13	142.89(14)
S(10)#5-Sr(2)-S(10)	68.28(18)	S(5)#10-Sr(6)-S(3)	130.11(15)
O(1)#4-Sr(2)-S(5)#5	75.41(17)	S(5)#10-Sr(6)-S(2)	138.56(16)
S(1)-Sr(2)-S(5)#5	136.68(14)	S(3)-Sr(6)-S(2)	76.73(15)
S(4)-Sr(2)-S(5)#5	67.77(13)	S(5)#10-Sr(6)-S(6)#10	75.92(14)
S(10)#5-Sr(2)-S(5)#5	71.08(13)	S(3)-Sr(6)-S(6)#10	119.91(14)
S(10)-Sr(2)-S(5)#5	135.65(13)	S(2)-Sr(6)-S(6)#10	121.84(14)
O(1)#4-Sr(2)-S(8)#2	66.6(5)	S(5)#10-Sr(6)-S(7)#1	78.52(14)
S(1)-Sr(2)-S(8)#2	97.56(14)	S(3)-Sr(6)-S(7)#1	149.21(16)
S(4)-Sr(2)-S(8)#2	98.78(14)	S(2)-Sr(6)-S(7)#1	73.03(14)
S(10)#5-Sr(2)-S(8)#2	136.03(14)	S(6)#10-Sr(6)-S(7)#1	73.24(14)
S(10)-Sr(2)-S(8)#2	134.84(14)	S(5)#10-Sr(6)-S(8)	76.51(14)
S(5)#5-Sr(2)-S(8)#2	70.06(13)	S(3)-Sr(6)-S(8)	74.67(13)
O(1)#4-Sr(2)-S(5)	72.93(16)	S(2)-Sr(6)-S(8)	83.79(14)
S(1)-Sr(2)-S(5)	66.19(13)	S(6)#10-Sr(6)-S(8)	151.70(15)
S(4)-Sr(2)-S(5)	136.03(13)	S(7)#1-Sr(6)-S(8)	107.22(15)
S(10)#5-Sr(2)-S(5)	133.28(14)	S(5)#10-Sr(6)-S(1)#10	68.49(14)
S(10)-Sr(2)-S(5)	69.01(13)	S(3)-Sr(6)-S(1)#10	74.54(14)
S(5)#5-Sr(2)-S(5)	136.31(19)	S(2)-Sr(6)-S(1)#10	150.45(14)
S(8)#2-Sr(2)-S(5)	70.12(13)	S(6)#10-Sr(6)-S(1)#10	68.76(13)
O(2)#1-Sr(3)-S(11)#7	103.5(3)	S(7)#1-Sr(6)-S(1)#10	134.29(14)
O(2)#1-Sr(3)-S(7)#1	70.1(3)	S(8)-Sr(6)-S(1)#10	95.15(15)
S(11)#7-Sr(3)-S(7)#1	172.75(18)	S(5)#10-Sr(6)-S(9)#10	143.63(15)
O(2)#1-Sr(3)-S(9)#8	145.0(3)	S(3)-Sr(6)-S(9)#10	68.00(12)
S(11)#7-Sr(3)-S(9)#8	81.57(16)	S(2)-Sr(6)-S(9)#10	69.89(13)
S(7)#1-Sr(3)-S(9)#8	105.62(14)	S(6)#10-Sr(6)-S(9)#10	68.14(12)

O(2)#1-Sr(3)-S(4)#9	70.2(3)	S(7)#1-Sr(6)-S(9)#10	95.87(14)
S(11)#7-Sr(3)-S(4)#9	108.14(12)	S(8)-Sr(6)-S(9)#10	138.06(13)
S(7)#1-Sr(3)-S(4)#9	73.40(14)	S(1)#10-Sr(6)-S(9)#10	92.81(14)
S(9)#8-Sr(3)-S(4)#9	75.28(13)	S(4)-Ge(1)-S(1)	110.59(19)
O(2)#1-Sr(3)-S(9)#10	144.1(3)	S(4)-Ge(1)-S(2)	113.2(2)
S(11)#7-Sr(3)-S(9)#10	80.53(16)	S(1)-Ge(1)-S(2)	108.93(19)
S(7)#1-Sr(3)-S(9)#10	102.55(14)	S(4)-Ge(1)-S(3)	107.7(2)
S(9)#8-Sr(3)-S(9)#10	70.70(18)	S(1)-Ge(1)-S(3)	102.44(19)
S(4)#9-Sr(3)-S(9)#10	143.22(16)	S(2)-Ge(1)-S(3)	113.5(2)
O(2)#1-Sr(3)-S(2)	72.5(3)	O(2)-Ge(2)-S(7)	107.9(4)
S(11)#7-Sr(3)-S(2)	104.47(12)	O(2)-Ge(2)-S(5)	104.1(4)
S(7)#1-Sr(3)-S(2)	70.67(13)	S(7)-Ge(2)-S(5)	115.4(2)
S(9)#8-Sr(3)-S(2)	140.55(15)	O(2)-Ge(2)-S(6)	104.8(4)
S(4)#9-Sr(3)-S(2)	134.91(14)	S(7)-Ge(2)-S(6)	112.1(2)
S(9)#10-Sr(3)-S(2)	71.97(13)	S(5)-Ge(2)-S(6)	111.5(2)
O(2)#12-Sr(4)-S(5)#12	67.2(3)	O(1)-Ge(3)-S(8)#3	104.97(17)
O(2)#12-Sr(4)-S(7)	73.8(3)	O(1)-Ge(3)-S(8)	104.97(17)
S(5)#12-Sr(4)-S(7)	76.96(14)	S(8)#3-Ge(3)-S(8)	113.57(14)
O(2)#12-Sr(4)-S(3)	133.0(3)	O(1)-Ge(3)-S(8)#1	104.97(17)
S(5)#12-Sr(4)-S(3)	123.08(14)	S(8)#3-Ge(3)-S(8)#1	113.57(14)
S(7)-Sr(4)-S(3)	149.52(15)	S(8)-Ge(3)-S(8)#1	113.57(14)
O(2)#12-Sr(4)-S(4)#10	67.1(3)	O(3)-Ge(4)-S(9)#3	108.61(17)
S(5)#12-Sr(4)-S(4)#10	67.14(13)	O(3)-Ge(4)-S(9)#1	108.61(17)
S(7)-Sr(4)-S(4)#10	134.42(14)	S(9)#3-Ge(4)-S(9)#1	110.32(16)
S(3)-Sr(4)-S(4)#10	75.93(13)	O(3)-Ge(4)-S(9)	108.61(17)
O(2)#12-Sr(4)-S(10)	70.4(3)	S(9)#3-Ge(4)-S(9)	110.32(16)
S(5)#12-Sr(4)-S(10)	134.18(14)	S(9)#1-Ge(4)-S(9)	110.32(16)
S(7)-Sr(4)-S(10)	75.25(13)	S(11)-Ge(5)-S(10)#5	113.05(17)
S(3)-Sr(4)-S(10)	98.43(14)	S(11)-Ge(5)-S(10)#6	113.05(17)
S(4)#10-Sr(4)-S(10)	110.86(14)	S(10)#5-Ge(5)-S(10)#6	105.67(19)
O(2)#12-Sr(4)-S(1)	144.9(3)	S(11)-Ge(5)-S(10)	113.05(17)
S(5)#12-Sr(4)-S(1)	133.32(15)	S(10)#5-Ge(5)-S(10)	105.67(19)
S(7)-Sr(4)-S(1)	83.46(13)	S(10)#6-Ge(5)-S(10)	105.66(19)
S(3)-Sr(4)-S(1)	66.07(13)		

Symmetry transformations used to generate equivalent atoms:

#1 -x+y,-x+1,z #2 -y+2/3,x-y+1/3,z+1/3 #3 -x+y+2/3,-x+4/3,z+1/3
#4 -y+1,x-y+1,z #5 x+2/3,y+1/3,z+1/3 #6 -x+y+1,-x+1,z
#7 -x+y+1,-x+2,z #8 -y+2,x-y+1,z #9 x-1/3,y-2/3,z-2/3
#10 x-2/3,y-1/3,z-1/3 #11 -x+y-1/3,-x+1/3,z-2/3 #12 -x+y+1/3,-x+2/3,z-1/3
#13 -y+1,x-y,z #14 -y+4/3,x-y+2/3,z-1/3 #15 x+1/3,y-1/3,z-1/3

#16 -y,x-y,z #17 -x+y,-x,z #18 -x+y+1/3,-x+5/3,z-1/3
#19 -y+1/3,x-y+2/3,z+2/3 #20 x+1/3,y+2/3,z+2/3 #21 -x+y+1/3,-x+2/3,z+2/3
#22 x-1/3,y+1/3,z+1/3

Table S3a. Atomic coordinates (× 10^4) and equivalent isotropic displacement parameters ($Å^2$ × 10^3) for $Sr_{18}Ge_9O_5S_{31}$ (enantiomer 1). U_{eq} is defined as one-third of the trace of the orthogonalized U_{ij} tensor.

Atom	*Wyck.*	x	y	z	U(eq)	BVS
Sr(1)	9b	-1454(1)	-71(1)	3043(1)	13(1)	1.91
Sr(2)	9b	-3431(1)	1956(1)	2916(1)	13(1)ss	2.07
Sr(3)	9b	-5118(1)	-3349(1)	2535(1)	15(1)	1.92
Sr(4)	9b	-1525(1)	1874(1)	1092(1)	12(1)	1.76
Sr(5)	9b	-239(1)	4671(1)	1047(1)	14(1)	1.90
Sr(6)	9b	-3212(1)	-1565(1)	1103(1)	14(1)	2.05
Ge(1)	9b	-3498(1)	-96(1)	2344(1)	10(1)	4.14
Ge(2)	9b	-114(1)	3212(1)	3125(1)	8(1)	3.96
Ge(3)	3a	0	0	1063(2)	8(1)	3.92
Ge(4)	3a	0	0	4374(2)	8(1)	4.04
Ge(5)	3a	-3333	3333	1176(2)	10(1)	4.17
O(1)	3a	0	0	71(12)	9(5)	2.20
O(2)	9b	814(8)	4161(8)	3564(7)	9(3)	1.94
O(3)	3a	0	0	3394(12)	9(5)	2.04
S(1)	9b	-2252(3)	1121(3)	2739(3)	10(1)	2.09
S(2)	9b	-3470(3)	-1332(3)	2708(3)	12(1)	2.04
S(3)	9b	-3398(3)	98(3)	1126(3)	11(1)	1.93
S(4)	9b	-4712(3)	-68(3)	2715(3)	13(1)	2.04
S(5)	9b	-1329(3)	3255(3)	3515(3)	11(1)	2.10
S(6)	9b	-82(3)	2001(3)	3568(3)	12(1)	1.87
S(7)	9b	124(3)	3365(3)	1919(3)	12(1)	2.14
S(8)	9b	-1221(3)	80(3)	1380(3)	14(1)	1.82
S(9)	9b	-1243(3)	-17(3)	4764(3)	13(1)	1.83
S(10)	9b	-2170(3)	3264(3)	1655(3)	16(1)	1.91
S(11)	3a	-3333	3333	-37(5)	15(2)	2.14

[a]Bond valence state was calculated using the empirical formula $V_i = \Sigma S_{ij} = \Sigma \exp[(r_0 - r_{ij})/0.37]$, where S_{ij} is the bond valence associated with bond lengths r_{ij} and r_0.

Table S3b. Atomic coordinates (× 10^4) and equivalent isotropic displacement parameters ($Å^2 \times 10^3$) for (enantiomer 2). U_{eq} is defined as one-third of the trace of the orthogonalized U_{ij} tensor.

Atom	*Wyck.*	x	y	z	U(eq)	BVS
Sr(1)	9b	4716(2)	6597(2)	6372(2)	13(1)	1.94
Sr(2)	9b	8718(2)	8622(2)	6249(2)	13(1)	2.06
Sr(3)	9b	1550(2)	1565(2)	2534(2)	17(1)	1.92
Sr(4)	9b	1810(2)	3267(2)	4424(2)	13(1)	1.78
Sr(5)	9b	8655(2)	6900(2)	4382(2)	14(1)	1.88
Sr(6)	9b	4979(2)	5091(2)	4433(2)	14(1)	2.16
Ge(1)	9b	6734(2)	6573(2)	5673(2)	8(1)	4.13
Ge(2)	9b	3446(2)	3440(2)	3126(2)	6(1)	3.91
Ge(3)	3a	3333	6667	4397(3)	6(1)	3.92
Ge(4)	3a	3333	6667	7708(3)	7(1)	3.82
Ge(5)	3a	0	0	4509(3)	6(1)	4.20
O(1)	3a	3333	6667	3411(15)	1(2)	2.21
O(2)	9b	2485(11)	2505(11)	3559(9)	4(4)	1.93
O(3)	3a	3333	6667	6691(15)	0(2)	1.94
S(1)	9b	6708(5)	7791(5)	6071(4)	11(2)	2.09
S(2)	9b	5472(5)	5343(4)	6036(4)	10(2)	2.05
S(3)	9b	6821(5)	6760(5)	4455(4)	13(2)	1.93
S(4)	9b	7981(4)	6602(5)	6044(4)	10(2)	2.02
S(5)	9b	3408(5)	4657(5)	3519(4)	14(2)	2.11
S(6)	9b	4663(5)	3409(5)	3571(4)	14(2)	1.86
S(7)	9b	3313(5)	3222(5)	1918(4)	10(2)	2.14
S(8)	9b	4635(5)	6744(5)	4718(4)	12(2)	1.81
S(9)	9b	3354(5)	7916(5)	8097(4)	10(2)	1.80
S(10)	9b	1164(5)	1234(5)	4985(4)	16(2)	1.90
S(11)	3a	0	0	3301(7)	17(3)	2.15

a)Bond valence state was calculated using the empirical formula $V_i = \Sigma S_{ij} = \Sigma \exp[(r_0 - r_{ij})/0.37]$, where S_{ij} is the bond valence associated with bond lengths r_{ij} and r_0.

Table S4. Properties comparison of $Sr_{18}Ge_9O_5S_{31}$ with other reported oxychalcogenides.

Number	Compound	SHG (×AGS)	E_g (eV)	IR cut-off edge (μm)
1	$Ba_2SnSSi_2O_7$[15]	0.005	2.70	-
2	$SrZn_2OS_2$[16]	0.06	3.86	-
3	CaZnOS[17]	0.25	3.71	-
4	$SrGeOS_2$[18]	0.40	3.90	-
5	$K_2Ba_{0.5}Ga_9O_2S_{13}$[19]	0.50	3.72	-

6	$BaGeOS_2$[18]	0.50	4.10	-
7	$Sr_2ZnGe_2OS_6$[20]	0.60	3.73	-
8	$Sr_2ZnSn_2OS_6$[21]	0.70	3.52	~12.5
9	$Ba_3S[GeOS_3]$[22]	0.70	3.63	~14
10	$Sr_2CdGe_2OS_6$[20]	0.80	3.62	~13.2
11	$Sr_3Ge_2O_4Se_3$[23]	0.80	2.96	-
12	$Nd_3[Ga_3O_3S_3][Ge_2O_7]$[24]	0.80	4.35	~13.7
13	$Sr_5Ga_8O_3S_{14}$[25]	0.80	3.90	~13.4
14	$LaCaGa_3S_6O$[26]	0.90	3.27	~14.8
15	$Sr_3[SnOSe_3][CO_3]$[27]	1.00	3.46	~7.0
16	$LaSrGa_3S_6O$[26]	1.00	3.21	~14.8
17	$BaGeOSe_2$[28]	1.10	3.20	~13
18	$Sr_3S[GeOS_3]$[22]	1.10	4.10	~14
19	$Ba_3Se[GeOSe_3]$[22]	1.20	3.52	~14
20	$SrGeOSe_2$[29]	1.30	3.16	~12.6
21	$Ba_3Ge_2O_4Te_3$[30]	1.50	2.10	~8
22	$Sr_3Se[GeOSe_3]$[22]	1.50	3.52	~14
23	$Sr_2GeGa_2OS_6$[31]	1.70	3.15	~13.4
24	$Ca_2GeGa_2OS_6$[31]	2.10	3.15	~13.4
25	$Sr_6Cd_2Sb_6O_7S_{10}$[32]	4.00	1.89	~15
This work	$Sr_{18}Ge_9O_5S_{31}$	2.50	3.61	~15.3

Table S5. Dipole moments (in Debye) of $[GeS_4]$ and $[GeOS_3]$ tetrahedra in the unit cell.

Compound	SBU	x	y	z	Dipole moment	net dipole moment
$Sr_{18}Ge_9O_5S_{31}$	Ge(1)S4	0	0	-3.77	3.77	8.24
	Ge(2)OS3	0	0	-2.41	2.41	
	Ge(3)OS3	0	0	3.44	3.44	
	Ge(4)OS3	0	0	7.32	7.32	
	Ge(5)S4	0	0	3.66	3.66	

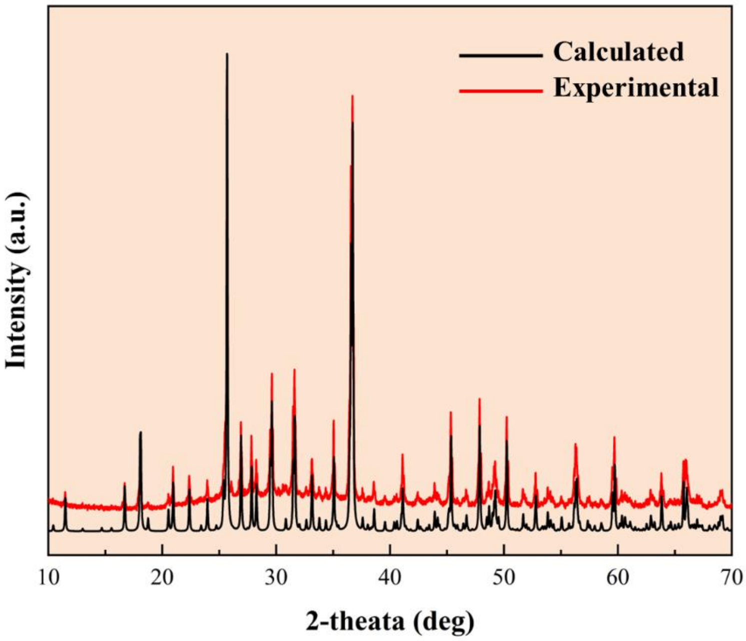

Figure S1. Calculated and experimental powder X-ray diffraction patterns of $Sr_{18}Ge_9O_5S_{31}$.

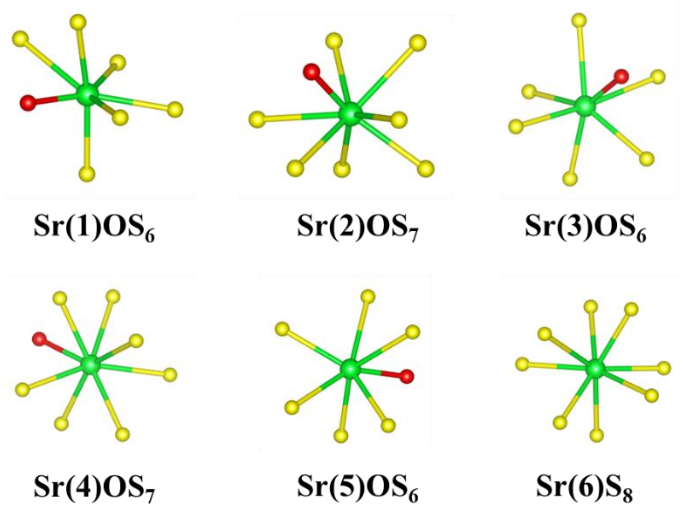

Figure S2. The coordination environment of Sr atoms.

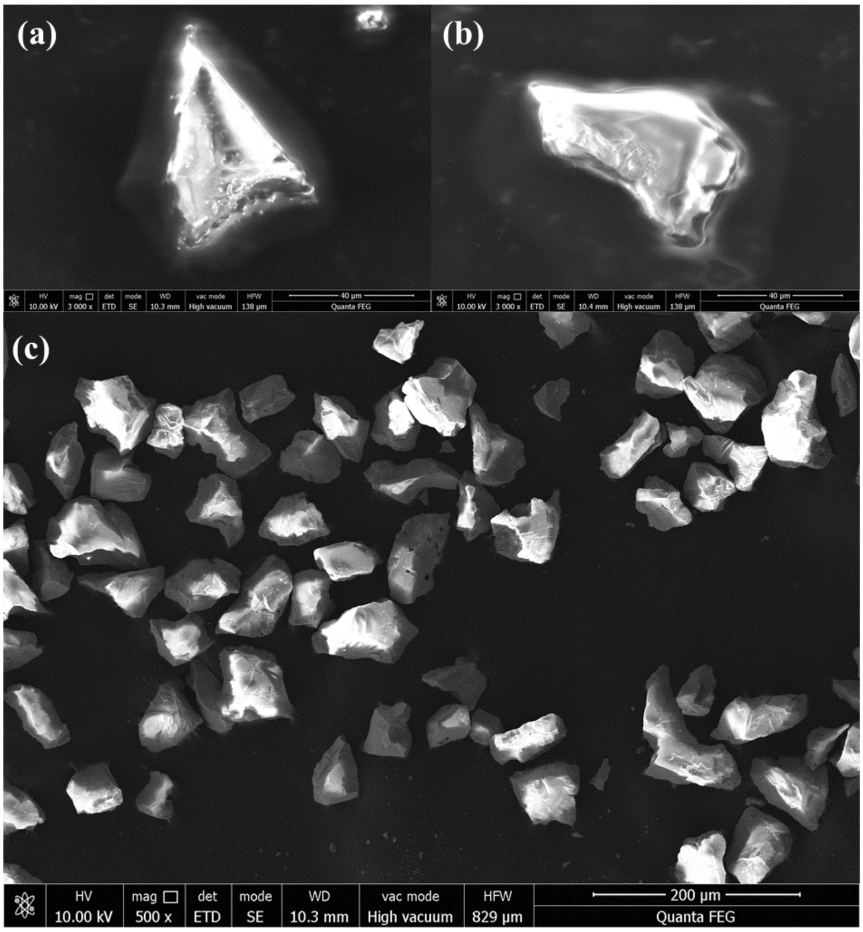

Figure S3. The morphology of (a) enantiomer 1, (b) enantiomer 2, and (c) polycrystalline for $Sr_{18}Ge_9O_5S_{31}$ captured by scanning electron microscopy (SEM) and no significant differences were found between the two enantiomers.

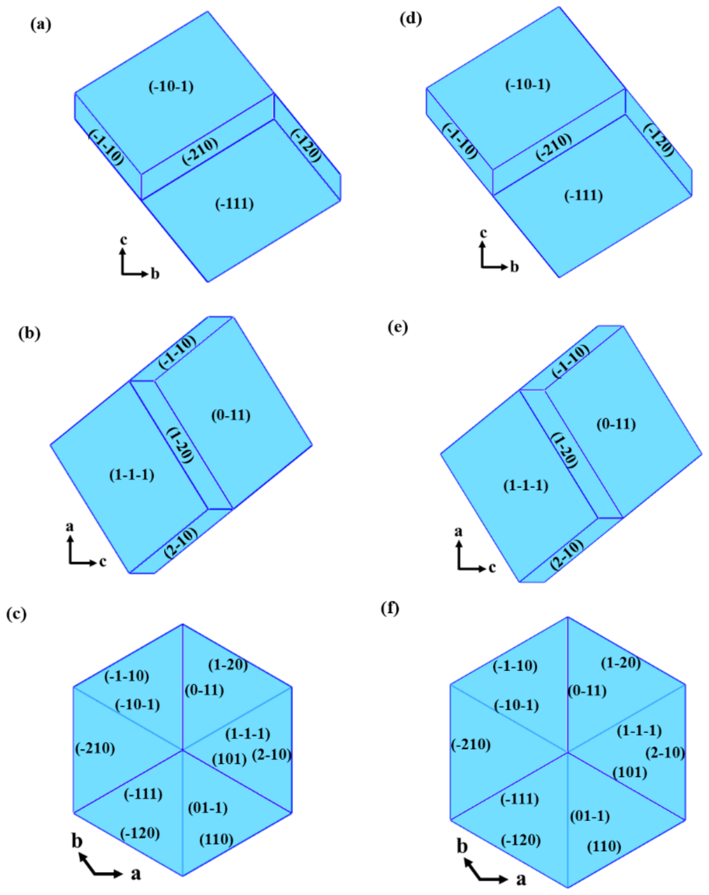

Figure S4. Theoretical morphology of enantiomer 1 (a-c) and enantiomer 2 (d-f) crystals calculated using Mercury program according to the Bravais-Friedel-Donnay-Harker (BFDH) theory.

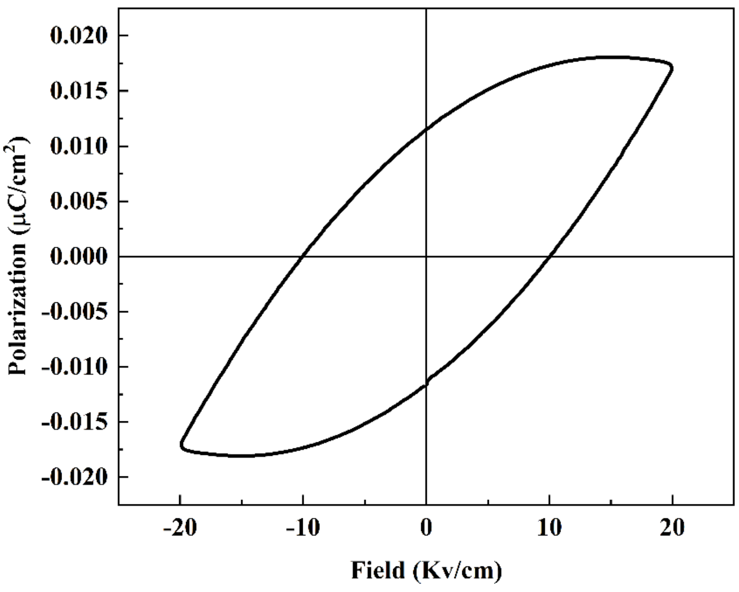

Figure S5. The polarization versus applied electric field (*P*-*E*) hysteresis curve of $Sr_{18}Ge_9O_5S_{31}$.

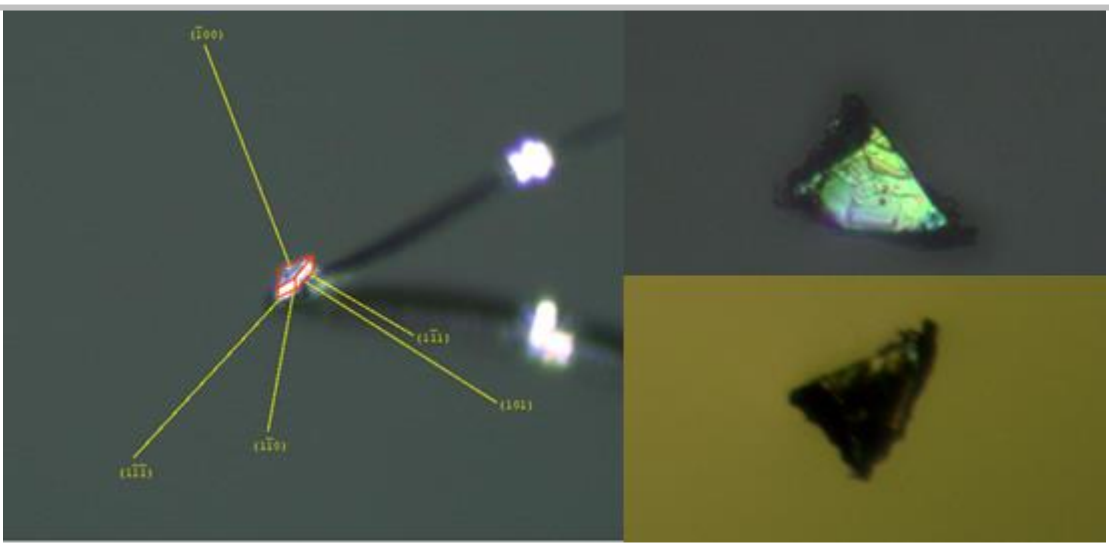

Figure S6. Crystal thickness picture of $Sr_{18}Ge_9O_5S_{31}$ for measuring of birefringence experiment and measured by cross-polarized light for $Sr_{18}Ge_9O_5S_{31}$ crystal.

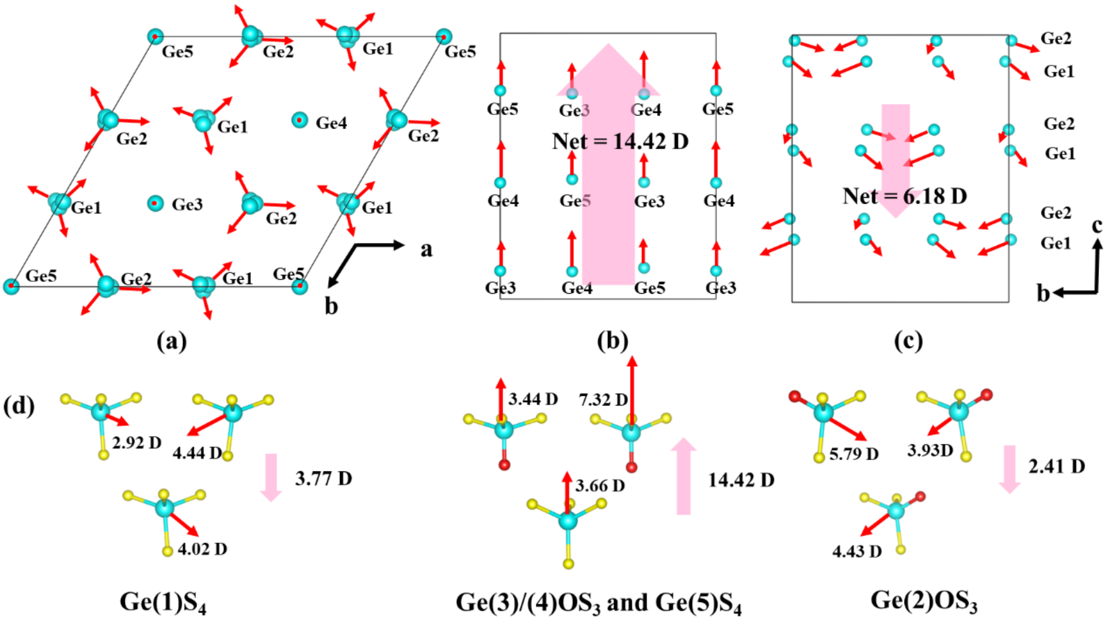

Figure S7. Schematic drawing of the direction and magnitude of the dipole moments for $Ge(1)S_4$, $Ge(2)OS_3$, $Ge(3)OS_3$, $Ge(4)OS_3$ and $Ge(5)S_4$ tetrahedra in the unit cell. The red arrows indicate the direction of the net dipole moment. All coordinated atoms and bonds in (a)-(c) were omitted for clarity.

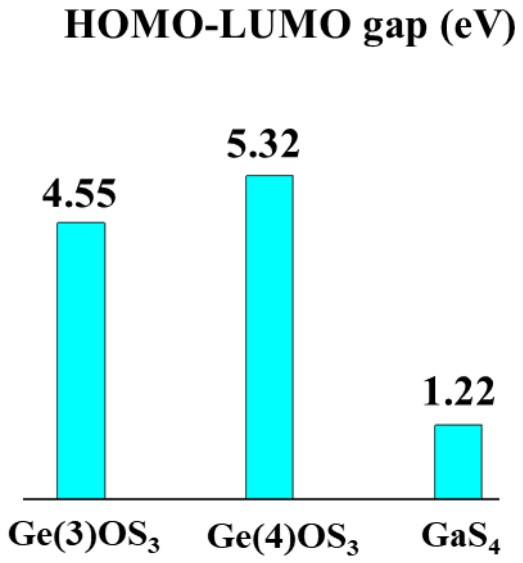

Figure S8. The HOMO−LUMO gaps of Ge(3)OS_3, Ge(4)OS_3, and GaS_{4s}.

References

[1] R. Masse, J. Grenier, A. Durif-Varambon, *Bull. Soc. Fr. Mineral. Cristallogr.* **1967**, *90*, 20-23.
[2] G. Blasse, *J. Inorg. Nucl. Chem.* **1968**, *30*, 2283-2284.
[3] O. V. Dolomanov, A. J. Blake, N. R. Champness, M. Schroder, *J. Appl. Crystallogr.* **2003**, *36*, 1283-1284.
[4] E. L. Simmons, *Appl. Opt.* **1975**, *14* 1380-1386.
[5] a) H. Yuan, Q. Gao, P. Xu, J. Guo, L. He, A. Sanson, M. Chao, E. Liang, *Inorg. Chem.* **2021**, *60*, 1499-1505; b) S. N. Achary, D. Errandonea, D. Santamaria-Perez, O. Gomis, S. J. Patwe, F. J. Manjon, P. R. Hernandez, A. Munoz, A. K. Tyagi, *Inorg. Chem.* **2015**, *54*, 6594-6605.
[6] a) K. Jackson, A. Briley, S. Grossman, *Phys. Rev. B* **1999**, *60*, 14985; b) Y. Kawamoto, C. Kawashima, *Mat. Res. Bull.* **1982**, *17*, 1511-1516.
[7] S. K. Kurtz, T. T. Perry, *J. Appl. Phys.* **1968**, *39*, 3798-3813.
[8] M. J. Zhang, X. M. Jiang, L. J. Zhou, G. C. Guo, *J. Mater. Chem. C.* **2013**, *1*, 4754-4760.
[9] S. J. Clark, M. Segall, C. J. Pickard, P. J. Hasnip, M. Probert, K. Refson, M. C. Payne, *Z. Krist. - Cryst. Mater.* **2005**, *220*, 567-570.
[10] J. P. Perdew, K. Burke, M. Ernzerhof, *Phys. Rev. Lett.* **1996**, *77*, 3865.
[11] J. S. Lin, A. Qteish, M. C. Payne, V. Heine, *Phys. Rev. B* **1993**, *47*, 4174-4180.
[12] a) S. Laksari, A. Chahed, N. Abbouni, O. Benhelal, B. Abbar, *Comput. Mater. Sci.* **2006**, *38*, 223-230; b) S. D. Mo, W. Y. Ching, *Phys. Rev. B. Condens. Mat.* **1995**, *51*, 13023-13032.
[13] C. Aversa, J. E. Sipe, *Phys. Rev. B.* **1995**, *52*, 14636.
[14] J. Lin, M. H. Lee, Z. P. Liu, C. Chen, C. J. Pickard, *Phys. Rev. B.* **1999**, *60*, 13380.
[15] B. Almoussawi, W. D. Yao, S. P. Guo, M. H. Whangbo, V. Dupray, S. Clevers, S. Deng, H. Kabbour, *Chem. Mater.* **2022**, *34*, 4375-4383.
[16] Y. Tsujimoto, C. A. Juillerat, W. Zhang, K. Fujii, M. Yashima, P. S. Halasyamani, H.-C. zur Loye, *Chem. Mater.* **2018**, *30*, 6486-6493.
[17] T. Sambrook, C. F. Smura, S. J. Clarke, *Inorg. Chem.* **2007**, *46*, 2571-2574.
[18] X. Zhang, Y. Xiao, R. Wang, P. Fu, C. Zheng, F. Huang, *Dalton Trans.* **2019**, *48*, 14662-14668.
[19] J.-N. Li, X.-H. Li, Y.-X. Xu, W. Liu, S.-P. Guo, *Chin. J. Chem.* **2022**, *40*, 2407-2414.
[20] M. Y. Ran, S. H. Zhou, B. Li, W. Wei, X. T. Wu, H. Lin, Q. L. Zhu, *Chem. Mater.* **2022**, *34*, 3853-3861.
[21] Y. Cheng, H. Wu, H. Yu, Z. Hu, J. Wang, Y. Wu, *Chem. Sci.* **2022**, *13*, 5305-5310.
[22] S. Cui, H. Wu, Z. Hu, J. Wang, Y. Wu, H. Yu, *Adv. Sci.* **2023**, *10*, e2204755.
[23] W. Xing, P. Fang, N. Wang, Z. Li, Z. Lin, J. Yao, W. Yin, B. Kang, *Inorg. Chem.* **2020**, *59*, 16716-16724.
[24] M. Y. Ran, S. H. Zhou, W. B. Wei, B. X. Li, X. T. Wu, H. Lin, Q. L. Zhu, *Small* **2023**, *19*, e2300248.
[25] R. Wang, Y. Guo, X. Zhang, Y. Xiao, J. Yao, F. Huang, *Inorg. Chem.* **2020**, *59*, 9944-9950.
[26] J. Xu, K. Wu, Y. Xiao, B. Zhang, H. Yu, H. Zhang, *ACS Appl. Mater. Inter.* **2022**, *14*, 37967-37974.
[27] J. Wang, Y. Cheng, H. Wu, Z. Hu, J. Wang, Y. Wu, H. Yu, *Angew. Chem. Int. Ed.* **2022**, *61*, e202201616.
[28] B. W. Liu, X. M. Jiang, G. E. Wang, H. Y. Zeng, M. J. Zhang, S. F. Li, W. H. Guo, G. C. Guo, *Chem. Mater.* **2015**, *27*, 8189-8192.
[29] M. Y. Ran, Z. Ma, H. Chen, B. X. Li, X. T. Wu, H. Lin, Q. L. Zhu, *Chem. Mater.* **2020**, *32*, 5890-5896.
[30] M. Sun, X. Zhang, C. Li, W. Liu, Z. Lin, J. Yao, *J. Mater. Chem. C* **2022**, *10*, 150-159.
[31] R. Wang, F. Liang, X. Liu, Y. Xiao, Q. Liu, X. Zhang, L.-M. Wu, L. Chen, F. Huang, *ACS Appl. Mater. Inter.* **2022**, *14*, 23645-23652.
[32] R. Wang, F. Liang, F. Wang, Y. Guo, X. Zhang, Y. Xiao, K. Bu, Z. Lin, J. Yao, T. Zhai, F. Huang, *Angew. Chem. Int. Ed.* **2019**, *58*, 8078-8081.
